# Comprehensive data of 5085 patients newly diagnosed with colorectal liver metastasis between 2013 and 2017: Fourth report of a nationwide survey in Japan

**DOI:** 10.1002/jhbp.12078

**Published:** 2024-11-12

**Authors:** Katsunori Sakamoto, Toru Beppu, Goro Honda, Kenjiro Kotake, Masakazu Yamamoto, Keiichi Takahashi, Itaru Endo, Kiyoshi Hasegawa, Michio Itabashi, Yojiro Hashiguchi, Yoshihito Kotera, Shin Kobayashi, Tatsuro Yamaguchi, Kazushige Kawai, Soichiro Natsume, Ken Tabuchi, Hirotoshi Kobayashi, Kensei Yamaguchi, Kimitaka Tani, Satoshi Morita, Yoichi Ajioka, Masaru Miyazaki, Kenichi Sugihara

**Affiliations:** ^1^ Joint Committee for Nationwide Survey on Colorectal Liver Metastasis; ^2^ Department of Surgery Graduate School of Medicine, Kyoto University 54 Kawahara‐Cho, Shogoin, Sakyo‐Ku Kyoto Japan; ^3^ Department of Surgery Yamaga City Medical Center 511 Yamaga Kumamoto Japan; ^4^ Department of Surgery Institute of Gastroenterology, Tokyo Women's Medical University 8‐1 Kawada‐Cho Shinjuku‐ku Tokyo Japan; ^5^ Department of Surgery Sano City Hospital Sano Tochigi Japan; ^6^ Department of Surgery Utsunomiya Memorial Hospital Utsunomiya Tochigi Japan; ^7^ Grace Home Care Clinic Ito Shizuoka Japan; ^8^ Department of Gastroenterological Surgery Yokohama City University Graduate School of Medicine Kanazawa‐ku Yokohama Kanagawa Japan; ^9^ Hepato‐Biliary‐Pancreatic Surgery Division, Department of Surgery Graduate School of Medicine, the University of Tokyo Bunkyo‐ku Tokyo Japan; ^10^ Saiseikai Kazo Hospital Kazo Saitama Japan; ^11^ Japanese Red Cross Omori Hospital Ota‐ku Tokyo Japan; ^12^ Department of Hepatobiliary‐Pancreatic Surgery Juntendo University Graduate School of Medicine Bunkyo‐ku Tokyo Japan; ^13^ Department of Hepatobiliary and Pancreatic Surgery National Cancer Center Hospital East Kashiwa‐shi Chiba Japan; ^14^ Department of Gastroenterology Tokyo Metropolitan Cancer and Infectious Diseases Center Komagome Hospital Bunkyo‐ku Tokyo Japan; ^15^ Department of Surgery Tokyo Metropolitan Cancer and Infectious Diseases Center Komagome Hospital Bunkyo‐ku Tokyo Japan; ^16^ Department of Pediatrics Tokyo Metropolitan Cancer and Infectious Diseases Center Komagome Hospital Bunkyo‐kuTokyo Japan; ^17^ Department of Surgery Teikyo University Hospital Takatsu‐ku Kawasaki Japan; ^18^ Department of Gastrointestinal Chemotherapy Cancer Institute Hospital of Japanese Foundation for Cancer Research Koto‐ku Tokyo Japan; ^19^ Department of Biomedical Statistics and Bioinformatics Graduate School of Medicine, Kyoto University Sakyo‐ku Kyoto Japan; ^20^ Division of Molecular and Diagnostic Pathology Niigata University Graduate School of Medical and Dental Sciences Chuo‐ku Niigata Japan; ^21^ International University of Health and Welfare, Narita Hospital Minato‐ku Tokyo Japan; ^22^ Tokyo Medical and Dental University Bunkyo‐ku Tokyo Japan

**Keywords:** colorectal neoplasm, liver neoplasm, neoplasm metastasis

## Abstract

The Joint Committee for Nationwide Survey on colorectal liver metastasis (CRLM) was established to improve treatment outcomes in patients with CRLM. The aim of this study was to evaluate the transition in the characteristics and treatment strategies of patients with CRLM and to analyze the prognostic factors. The data of 5085 patients newly diagnosed between 2013 and 2017 were compared with those of 3820 patients from 2005 and 2007. In patients who underwent hepatectomy (*n* = 2759 and 2163), the number of CRLMs was significantly higher and in the 2013–2017 data than in the 2005–2007 data (median 2 vs. 1; *p* = .005). Overall survival (OS) rates after diagnosis of CRLM after hepatectomy were better in the 2013–2017 data than that in the 2005–2007 data (5‐year OS, 62.4% vs. 56.7%, *p* < .001). Recurrence‐free survival (RFS) after hepatectomy was comparable between the groups (5‐year RFS, 30.5% vs. 30.7%; *p* = .068). Multivariate analyses identified age at diagnosis of CRLM ≥70 years, lymph node metastasis of primary lesion, preoperative carbohydrate antigen (CA) 19–9 value >100 U/mL, number of CRLM 2–4, and R2 resection as independent predictors of OS. Synchronous CRLM, concomitant extrahepatic metastasis, lymphatic invasion, lymph node metastasis of primary lesion, preoperative CA19‐9 value >100 U/mL, number of CRLM 5–, and nonlaparoscopic approach were selected as that of RFS. Despite having a higher prevalence of advanced stage CRLM in the 2013–2017 patient population compared to the 2005–2007 cohort, prognostic outcomes demonstrably improved in the later period.

## Graphical abstract

Sakamoto and colleagues analyzed data from the Joint Committee for Nationwide Survey on Colorectal Liver Metastasis on 5085 patients newly diagnosed with colorectal liver metastasis between 2013 and 2017. Despite a higher prevalence of advanced disease, patient outcomes during this period demonstrably improved compared with those of the 2005–2007 cohort.

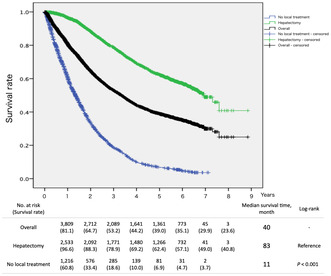

## 1 INTRODUCTION

The liver is the most frequent location of metastatic tumors derived from colorectal cancer.^1^, ^2^, ^3^, ^4^ To date, resection is the only curative treatment for patients with colorectal liver metastasis (CRLM).^1^ The prognosis of patients with CRLM has shown improvement, with reported 5‐year overall survival (OS) approaching 60% in post‐hepatectomy patients.^1^, ^3^, ^5^ However, 70%–80% of tumors are considered unresectable at initial presentation, and prognosis is poor in patients who do not undergo hepatectomy.^1^ Multidisciplinary treatment is required for marginally resectable or unresectable CRLM, and advances in surgical procedures, perioperative management, and chemotherapy have contributed to improved prognosis in these patients.^1^ To further improve treatment outcomes in patients with CRLM, large‐scale data are required. The Joint Committee for Nationwide Survey on CRLM was established by the Japanese Society for Cancer of the Colon and Rectum (JSCCR) and the Japanese Society of Hepato‐Biliary‐Pancreatic Surgery (JSHBPS). The Joint Committee will provide raw anonymized data to researchers to perform studies that meet their aims as previously described.^6^, ^7^, ^8^, ^9^

The Joint Committee retrospectively collected the data of patients who were diagnosed with CRLM between 2005 and 2007 in 2014,^5^ and the data of patients newly diagnosed with CRLM after 2013 were continuously and prospectively registered.^5^, ^10^, ^11^ A follow‐up period of up to 5 years was achieved for patients diagnosed with CRLM from 2013 to 2017. Therefore, this study aimed to show representative data of patients newly diagnosed with CRLM between 2013 and 2017 and compare the transition in characteristics, treatment strategies, and prognostic outcomes with those of patients newly diagnosed with CRLM between 2005 and 2007.

## 2 METHODS

Among the institutions participating in the JSCCR and specially qualified board‐certified training institutions or departments certified by the JSHBPS,^12^ 209 departments from 201 institutions agreed to participate in this nationwide database system. All the data were registered using the original comprehensive database system created by the Joint Committee.

To protect personal information, all data were registered using a linkable anonymous code that could be connected to and used only at each institution before registration. The registered data were rigorously managed by reducing and excluding data with deficiencies in six fundamental items (age, sex, verified latest date of survival, prognosis, number of liver metastases, and maximum size of liver tumor) and by checking and integrating duplicate registrations at the registration secretariat of the committee to maintain data quality.

A total of 5949 patients were newly diagnosed with CRLM between 2013 and 2017. After conducting a quality management process, the present report summarizes the data of 5085 patients in terms of patient characteristics, clinical findings associated with CRLM at the time of diagnosis, treatment strategies, postoperative outcomes of hepatectomy, time‐series data on tumor markers, clinicopathological findings of the primary lesion, implementation status of chemotherapy, and prognostic data. Data of 5085 patients were compared with those of 3820 patients newly diagnosed with CRLM between 2005 and 2007. The prognosis of the data from 2013 to 2017 was compared with that of 2005–2007 according to the classification of CRLM using the General Rules for Clinical and Pathological Studies on Cancer of the Colon, Rectum, and Anus (seventh edition, revised version, January 2009)^13^: H1 (1 to 4 metastatic tumors, all of which are 5 cm or less in maximum diameter), H2 (other than H1 or H3), and H3 (5 or more metastatic tumors, at least one of which is more than 5 cm in maximum diameter). Prognostic data were collected in 2014 for 2005–2007, and those for 2013, 2014, 2015, 2016, and 2017 were collected in 2019, 2020, 2021, 2022, and 2023, respectively. The median follow‐up period was 29 months (interquartile range, 13–65 months) for the 2005–2007 data, and 26 months (interquartile range, 11–62 months) for the 2013–2017 data.

The prognostic factors associated with OS and RFS were analyzed by univariate and multivariate analyses in 2759 patients who underwent hepatectomy alone as local treatment for CRLM and were newly diagnosed with CRLM between 2013 and 2017. Patients who underwent R2 resection were included in the OS analyses but were excluded from the RFS analyses.

### 2.1 Statistical analysis

Continuous variables are presented as medians and quartiles, and nominal and ordinal variables are presented as ratios. Local treatment for CRLM includes hepatectomy and ablation therapy (radiofrequency ablation and microwave coagulation therapy) but excludes hepatic arterial infusion chemotherapy. Survival curves were generated using the Kaplan–Meier method and compared using the log‐rank test. Multivariate analyses were performed using a Cox proportional hazards model. For the multivariate analyses, the data were screened for multicollinearity. Values of *p* < .05 were considered to indicate statistical significance. All statistical analyses were performed using the SPSS software (version 24.0; IBM Corp., Armonk, NY, USA).

### 2.2 Ethical considerations

This study was approved by the Institutional Review Board of each institution (approval no. 1168: Tokyo Metropolitan Cancer and Infectious Diseases Center Komagome Hospital; data analyzed at this institute) and was performed in accordance with the ethical standards laid down in an appropriate version of the Declaration of Helsinki in 1995 (as revised in Brazil in 2013). Informed consent was obtained on the basis of the opt‐out principle. The details of the study and right to refuse to participate were disclosed online to the public.

## 3 RESULTS

### 3.1 Clinical characteristics of patients who underwent hepatectomy

The patients who underwent concomitant ablation therapy as local treatment for CRLM (*n* = 29) were excluded from the analyses. The age of registered patients was significantly higher in the 2013–2017 data than in the 2005–2007 data (median 66‐year‐old vs. 64‐year‐old, *p* < .001, Table 1), but sex showed no significant difference (male sex 62.5% vs. 63.2%, *p* = .626). The proportion of the patients with synchronous CRLM and primary lesions was significantly higher in the 2013–2017 data than in the 2005–2007 data (55.1% vs. 49.8%, *p* < .001; Table 1). The number of CRLMs was significantly higher in the 2013–2017 data compared with the 2005–2007 data (median 2 vs. 1; *p* = .005; Table 2). In contrast, the maximum diameter of the CRLM was significantly smaller in the 2013–2017 data than in the 2005–2007 data (median: 24 mm vs. 26 mm, *p* < .001; Table 2). The incidence of marginally resectable/initially unresectable CRLM was significantly higher in the 2013–2017 data than in the 2005–2007 data (6.5%/6.5%, 5.0%/4.1%; *p* < .001; Table 2). The JSHBPS nomogram score was significantly higher in the 2013–2017 data than in the 2005–2007 data (median, 7 vs. 6, *p* < .001; Table 2). Regarding the operative findings (Table 3), the operative time was significantly longer in the 2013–2017 data compared with the 2005–2007 data (median 320 min vs. 289 min, *p* < .001), whereas the amount of blood loss was significantly lower in the 2013–2017 data compared with the 2005–2007 data (median 320 mL vs. 550 mL, *p* < .001). The incidence of red blood cell transfusion was not significantly different between the groups (19.9% vs. 21.7%, *p* = .145). The proportion of patients who underwent neoadjuvant chemotherapy and laparoscopic surgery was significantly higher in the 2013–2017 data than those in the 2005–2007 data (20.8% vs. 5.2% and 26.1% vs. 2.3%, both *p* < .001, Table 3). Surgical curability showed no significant difference between both groups (*p* = .163). The other data associated with patients newly diagnosed with CRLM between 2013 and 2017 are presented in Data S9.

**TABLE 1:** Patient characteristics who underwent hepatectomy for CRLM^a^.

		2013–2017	2005–2007	*p*‐value
		*n* = 2759	*n* = 2163	
Age, years	Median (25, 75%)	66 (59, 73)	64 (57, 71)	<.001
Sex	Male	1725 (62.5%)	1367 (63.2%)	.626
Height, cm	Median (25, 75%)	161.6 (155.0, 168.0)	161.0 (154.0, 167.5)	.009
	Missing	55	434	
Weight, kg	Median (25, 75%)	57.8 (50.0, 66.0)	57.0 (50.0, 65.0)	.045
	Missing	57	432	
BMI	Median (25, 75%)	22.10 (19.82, 24.45)	22.21 (20.06, 24.28)	.924
	Missing	57	436	
HBs‐Ag	Positive	55 (2.1%)	35 (2.0%)	.799
	Missing	156	411	
HCV‐Ab	Positive	80 (3.1%)	59 (3.4%)	.593
	Missing	159	419	
Emergence time of CRLM^b^	Synchronous	1521 (55.1%)	1074 (49.7%)	<.001
	Metachronous	1238 (44.9%)	1089 (50.3%)	
Resection of primary lesion	Yes	2729 (98.9%)	2150 (99.4%)	.069

Abbreviations: BMI, body mass index; CRLM, colorectal liver metastasis; HBs‐Ag, hepatitis B virus surface antigen; HCV‐Ab, hepatitis C virus antibody.

^a^ Patients who underwent concomitant ablation therapy were excluded.

^b^ Synchronous liver metastasis was defined as a metastatic liver tumor diagnosed in the period of preoperative examination or surgery for primary lesion.

**TABLE 2:** Clinical findings of CRLM at diagnosis of patients who underwent hepatectomy for CRLM^a^.

		2013–2017	2005–2007	*p*‐value
		*n* = 2759	*n* = 2163	
Number of CRLMs	Median (25, 75%)	2 (1, 3)	1 (1, 3)	.005
Maximum diameter of CRLM, mm	Median (25, 75%)	24 (15, 38)	26 (17, 40)	<.001
Distribution of CRLMs	Bilobar	933 (34.0%)	678 (31.8%)	.114
	Unilobar	1813 (66.0)	1452 (68.2%)	
	Missing	13	33	
Local treatability on clinical findings^b^	Treatable	2397 (87.0%)	1874 (90.9%)	<.001
	Marginally treatable	179 (6.5%)	104 (5.0%)	
	Initially untreatable	179 (6.5%)	84 (4.1%)	
	Missing	4	101	
Concomitant extrahepatic metastasis	Yes	340 (12.4%)	282 (13.5%)	.260
	Missing	14	71	
Preoperative albumin, g/dL	Median (25, 75)	4.0 (3.8, 4.3)	4.1 (3.8, 4.4)	<.001
	Missing	140	321	
Preoperative T‐Bil, mg/dL	Median (25, 75)	0.6 (0.5, 0.8)	0.7 (0.5, 0.9)	<.001
	Missing	131	364	
Preoperative prothrombin time, %	Median (25, 75)	99.2 (89.3, 107.0)	97.0 (86.7, 103.0)	<.001
	Missing	195	437	
Preoperative ICG‐R15, %	Median (25, 75)	9.0 (5.7, 13.0)	8.0 (5.2, 11.8)	<.001
	Missing	655	833	
Preoperative hepatic coma	Yes	1 (0.0%)	1 (0.1%)	.644
	Missing	265	475	
Preoperative ascites	Yes	42 (1.7%)	15 (0.9%)	.030
	Missing	262	475	
JSHBPS nomogram score^c^	Median (25, 75)	7 (3, 10)	6 (3, 10)	<.001
JSHBPS nomogram risk score^c^	Low risk (−6)	1284 (46.5%)	1113 (51.5%)	<.001
	Moderate risk (7–10)	817 (29.6%)	623 (28.8%)	
	High risk (11–)	658 (23.8%)	427 (19.7%)	

Abbreviations: ASA, American Society of Anesthesiologists; CRLM, colorectal liver metastasis; JSHBPS, Japanese Society of Hepato‐Biliary‐Pancreatic Surgery; ICG‐R15, Indocyanine green retention rate at 15 minutes; T‐Bil, total bilirubin.

^a^ Patients who underwent concomitant ablation therapy were excluded.

^b^ Treatable: All tumors could be removed with preservation of a negative surgical margin and sufficient remnant liver volume without performing two‐stage hepatectomy with portal vein embolization or major vessel reconstruction. Marginally treatable: All tumors could be removed with preservation of remnant liver volume, regardless of surgical margin, in two‐stage hepatectomy with portal vein embolization or major vessel reconstruction. Untreatable: All tumors could not be removed with preservation of sufficient remnant liver volume even if two‐stage hepatectomy with portal vein embolization or major vessel reconstruction was performed. Lesions that could be completely ablated were included in the “treatable” category.

^c^ Calculated using the report from Beppu et al.^12^

**TABLE 3:** Operative outcomes of patients who underwent hepatectomy for CRLM^a^.

		2013–2017	2005–2007	*p*‐value
		*n* = 2759	*n* = 2163	
Operation time^b^, min	Median (25, 75%)	320 (229, 433)	289 (210, 384)	<.001
	Missing	79	245	
Amount of blood loss, mL	Median (25, 75%)	320 (120, 700)	550 (270, 1031)	<.001
	Missing	258	268	
Red blood cell transfusion	Yes	517 (19.9%)	385 (21.7%)	.145
	Missing	159	389	
Final pathological number of CRLMs	Median (25, 75%)	2 (1, 3)	1 (1, 3)	.002
	Missing	40	85	
Procedure of hepatectomy	Hr2 or Hr3	14 (0.6%)	78 (3.8%)	<.001
	Hr1	614 (25.3%)	389 (19.2%)	
	HrS	268 (11.0%)	139 (6.8%)	
	Hr0	1535 (63.1%)	1425 (70.2%)	
	Missing	328	132	
Pathological surgical margin, mm	Median (25, 75%)	3 (1, 9)	5 (1, 10)	.043
	Missing	1140	1187	
Surgical curability^c^	R0	2462 (91.4%)	1876 (93.0%)	.163
	R1	183 (6.8%)	105 (5.2%)	
	R2	43 (1.6%)	33 (1.6%)	
	RX	6 (0.2%)	4 (0.2%)	
	Missing	65	145	
Neoadjuvant chemotherapy	Yes	574 (20.8%)	112 (5.2%)	<.001
Simultaneous resection of primary lesion and CRLM in synchronous metastasis patients	Yes	560 (20.0%)	603 (27.9%)	<.001
	Missing	5	6	
Laparoscopic surgery	Total	685 (26.1%)	47 (2.3%)	<.001
	HALS	28 (1.1%)	5 (0.2%)	<.001
	Hybrid	44 (1.7%)	15 (0.7%)	
	Pure	613 (23.3%)	27 (1.3%)	
	Missing	125	100	
Preoperative portal vein embolization	Yes	463 (16.8%)	203 (9.4%)	<.001
Two‐stage hepatectomy	Yes	40 (1.4%)	7 (0.3%)	<.001
Residual extrahepatic lesion	Yes	211 (7.8%)	132 (6.1%)	.023
	Missing	62	12	
Hilar lymph node dissection	Yes	68 (2.6%)	66 (3.4%)	.116
	Missing	138	215	
Pathological hilar lymph node metastasis	Yes	28 (1.4%)	24 (1.9%)	.266
	Missing	766	905	
Pathological effect of chemotherapy^d^	Grade 0	69/666 (10.4%)^e^	33/181 (18.2%)^f^	.027
	Grade 1	355/666 (53.3%)^e^	93/181 (51.4%)^f^	
	Grade 2	189/666 (28.4%)^e^	41/181 (22.7%)^f^	
	Grade 3	53/666 (8.0%)^e^	14/181 (7.7%)^f^	
Postoperative complication (Clavien‐Dindo classification ≥ III)	Yes	293 (10.8%)	272 (13.3%)	.007
	Missing	36	116	
Postoperative hospital stays, day	Median (25, 75%)	12 (9, 16)	14 (10, 21)	<.001
	Missing	216	953	
Postoperative in‐hospital mortality	Yes	16 (0.6%)	29 (1.4%)	.005
	Mortality within 30 postoperative days	1 (0.0%)	2 (0.1%)	.410
	Mortality within 90 postoperative days	7 (0.3%)	9 (0.4%)	.321

*Note*: Neoadjuvant chemotherapy was performed in 623 of all CRLM patients, and of 623, 574 patients underwent hepatectomy. Neoadjuvant chemotherapy was performed in 130 of all CRLM patients, and of 130, 112 patients underwent hepatectomy.

Abbreviations: CRLM, colorectal liver metastasis; Hr0, partial hepatectomy; HrS, resection of one segment; Hr1, resection of one section; Hr2, resection of two sections; Hr3, resection of three sections; HALS, hand‐assisted laparoscopic surgery; Hybrid, hybrid laparoscopic surgery; Pure, pure laparoscopic surgery.

^a^ Patients who underwent concomitant ablation therapy were excluded.

^b^ If simultaneous resection of the primary lesion and CRLM was undertaken in patients with synchronous metastasis, the operation time was the sum of the duration of each procedure.

^c^ R0, no residual tumor; R1, microscopic residual tumor; R2, macroscopic residual tumor; RX, presence of residual tumor cannot be assessed.

^d^ Grade 0, no change, no necrosis or cellular or structural change can be seen throughout the lesion; Grade 1, mild change, necrosis, or disappearance of the tumor is present in less than two‐thirds of the whole lesion; Grade 2, moderate change, necrosis, or disappearance of the tumor is present in more than two‐thirds of the whole lesion, but viable tumor cells still remain; Grade 3, severe change, the whole lesion shows necrosis, and no viable tumor cells are observed.

^e^ Of the 1012 patients who underwent chemotherapy before local treatment, the data of 346 patients were missing (final total, *n* = 666).

^f^ Of the 468 patients who underwent chemotherapy before local treatment, the data of 287 patients were missing (final total, *n* = 181).

### 3.2 Survival analyses

**FIGURE 1:**
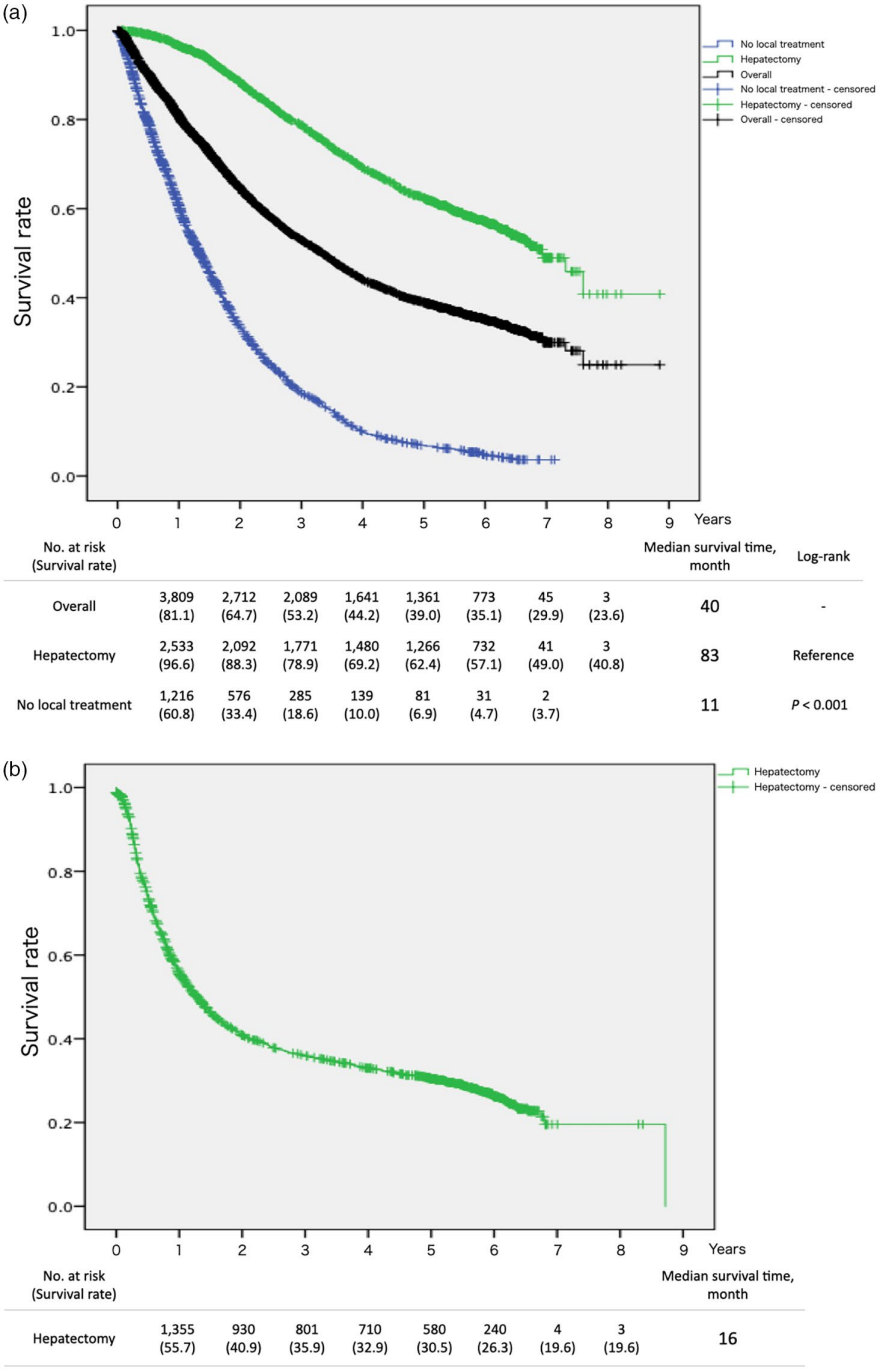
Overall survival and recurrence‐free survival of patients newly diagnosed with colorectal liver metastasis between 2013 and 2017. The patients who underwent concomitant local ablation therapy were excluded from the group of hepatectomy. (a) Overall survival after diagnosis. Overall group includes the patients who underwent local ablation therapy (with/without hepatectomy). (b) Recurrence‐free survival after hepatectomy.

**FIGURE 2:**
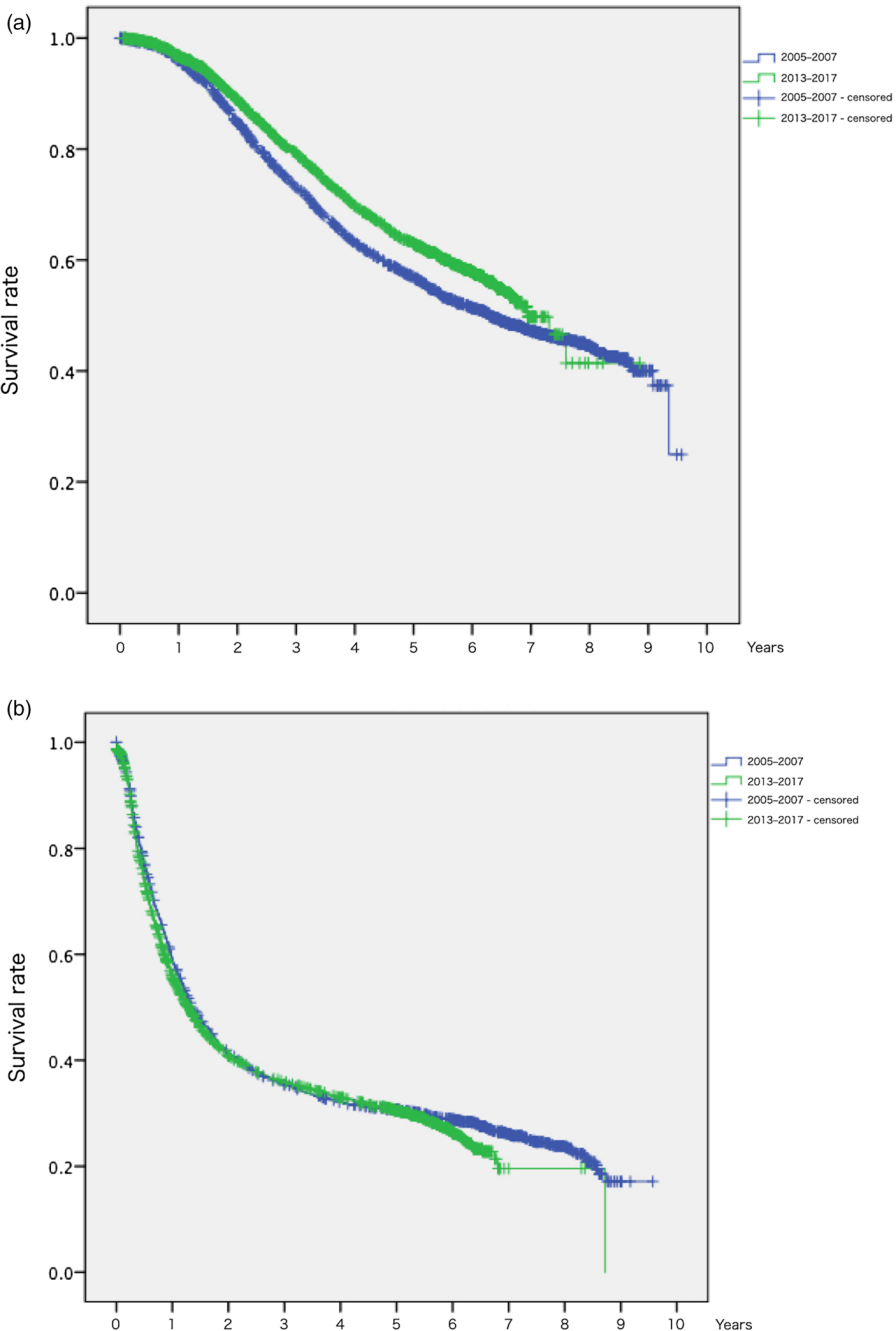
Comparison of survival among patients who underwent hepatectomy between 2013 and 2017 data and 2005 and 2007 data. (a) Overall survival after diagnosis. One‐, 3‐, and 5‐year overall survival rates were 96.6%, 78.9% and 62.4% in 2013–2017 data (*n* = 2759, median survival time 83 months) versus 95.6%, 72.7% and 56.6% in 2005–2007 data (*n* = 2163, median survival time 75 months) (*p* < .001). (b) Recurrence‐free survival after hepatectomy. One‐, 3‐, and 5‐year recurrence‐free survival rates were 55.7%, 35.9%, and 30.5% in 2013–2017 data (*n* = 2589, median survival time 16 months) versus 59.2%, 35.4%, and 30.7% in 2005–2007 data (*n* = 1985, median survival time 16 months) (*p* = .068).

The overall 1‐, 3‐, and 5‐year survival (OS) rates after the diagnosis of CRLM in patients who underwent hepatectomy alone as local treatment for CRLM in the 2013–2017 data were 96.6%, 78.9%, and 62.4%, respectively (Figure 1a). The 1‐, 3‐, and 5‐year recurrence‐free survival (RFS) rates after hepatectomy in the patients who underwent hepatectomy alone as local treatment for CRLM between in the 2013–2017 data were 55.7%, 35.9%, and 30.5%, respectively (Figure 1b). In the patients who underwent hepatectomy, compared with the 2005–2007 data, OS rates was better in the 2013–2017 data (Figure 2a, 5‐year OS 63.1% vs. 56.9%, *p* < .001), whereas RFS rates showed no significant differences between the groups (Figure 2b, 5‐year RFS 30.5% vs. 30.7%, *p* = .068).

According to the classification of CRLM using the General Rules for Clinical and Pathological Studies on Cancer of the Colon, Rectum, and Anus (seventh edition, revised version, January 2009), although OS in 2013–2017 data in H1 and H2 showed significantly better prognosis compared with 2005–2007 data (H1, 5‐year OS 65.2% vs. 60.4%, *p* = .014; H2, 56.0% vs. 46.8%, *p* < .001; Data S1 and S2), OS in 2013–2017 data in H3 showed no significant difference compared with 2005–2007 data (5‐year OS 44.4% vs. 38.9%, *p* = .182; Data S3). RFS in all H1–H3 classifications showed no significant differences between the two groups (H1, 5‐year RFS 34.8% vs. 34.0%, *p* = .233; H2, 5‐year RFS 18.6% vs. 21.9%, *p* = .190; H3, 5‐year RFS 8.2% vs. 8.2%, *p* = .673, Data [Link], [Link], [Link]).

According to the classification of JSHBPS nomogram risk score,^14^ both the OS and RFS were clearly stratified (5‐year OS 71.8% in low‐risk vs. 57.0% in moderate‐risk vs. 51.1% in high‐risk; 5‐year RFS were 45.2% in low‐risk vs. 22.2% in moderate‐risk vs. 13.9% in high‐risk; Data S7 and S8).

### 3.3 Prognostic factors of the 2013–2017 data in the patients underwent hepatectomy

Survival analyses of OS and RFS in patients who underwent hepatectomy alone as local treatment for CRLM using 2013 the 2017 data are presented in Tables 4 and 5. Preoperative chemotherapy included patients who underwent either neoadjuvant or induction therapy. Although survival rates were significantly lower in patients who underwent concomitant ablation therapy with hepatectomy and in those who had pathological hepatic hilar lymph node metastasis than in those who did not, these patients were excluded from multivariate analyses because of the small sample size. Multivariate analyses identified age at diagnosis of CRLM ≥70 years, lymph node metastasis of primary lesion, preoperative carbohydrate antigen (CA) 19–9 value >100 U/mL, number of CRLM 2–4, and R2 resection as independent predictors of OS (Table 4); and identified synchronous CRLM, concomitant extrahepatic metastasis, lymphatic invasion, lymph node metastasis of primary lesion, preoperative CA19‐9 value >100 U/mL, number of CRLM 5–, and nonlaparoscopic approach were selected as independent predictors of RFS (Table 5).

**TABLE 4:** Uni‐ and multivariate analyses for overall survival after the diagnosis of CRLM in patients who underwent hepatectomy using the data of 2013–2017.

		*n*	Univariate analysis^a^				Multivariate analysis			
			3‐y	5‐y	*p*‐value	*p*‐value	Hazard ratio	95% CI		*p*‐value
Patient characteristics										
Age, years	<70	1358	83.2	66.8	Reference	‐	Reference			
	≥70	1401	74.6	58.1	<.001	‐	1.468	1.114	1.934	.006
Sex	Female	1034	78.9	63.7	Reference	‐	NA			
	Male	1725	78.9	61.7	.168	‐				
Body mass index	≥25	2141	77.8	61.4	Reference	‐	NA			
	<25	558	82.8	65.6	.323	‐				
Preoperative MRI	Yes	1058	76.9	63.2	Reference	‐	NA			
	No	1656	80.1	61.8	.858	‐				
Preoperative chemotherapy^b^	No	956	81.5	66.6	Reference	‐	Reference			
	Yes	1012	78.5	59.0	.003	‐	1.435	0.897	2.295	.132
Emergence time of CRLM	Metachronous	1238	81.1	66.1	Reference	‐	Reference			
	Synchronous	1521	77.1	59.5	<.001	‐	1.164	0.823	1.645	.390
Concomitant extrahepatic metastasis	No	2405	80.3	64.5	Reference	‐	Reference			
	Yes	340	69.5	48.6	<.001	‐	1.382	0.971	1.967	.072
Characteristics of primary lesion										
Location of primary lesion	Left side	1982	80.7	64.9	Reference	‐	Reference			
	Right side	724	74.2	56.9	<.001	‐	1.192	0.872	1.628	.270
Pathological tumor depth of primary lesion^c^	T1/T2	237	84.8	71.7	Reference	‐	Reference			
	T3/T4	2423	78.5	61.8	.004	‐	1.338	0.787	2.274	.282
Lymphatic invasion^c^	ly0/1	2036	80.9	64.9	Reference	‐	Reference			
	ly2/3	626	71.6	54.1	<.001	‐	1.230	0.878	1.723	.229
Venous invasion^c^	v0/1	1439	80.9	65.4	Reference	‐	Reference			
	v2/3	1206	76.0	59.1	.002	‐	1.088	0.810	1.461	.575
Lymph node metastasis^c^	N0	887	85.5	73.0	Reference	‐	Reference			
	N1	1080	80.0	60.6	<.001	Reference	1.467	1.036	2.078	.031
	N2/3	678	69.2	52.6	<.001	< 0.001	1.636	1.103	2.426	.014
Histological type^c^	tub1	703	82.1	66.2	Reference	‐	Reference			
	tub2	1842	78.4	62.0	.015	Reference	0.897	0.444	1.813	.762
	Others	147	66.8	51.7	<.001	0.022	1.113	0.580	2.133	.748
*KRAS* status	Wild	563	78.4	58.7	Reference	‐	Reference			
	Mutant	893	69.7	49.9	.004	‐	1.185	0.887	1.584	.250
Tumor marker value										
CA19‐9, U/mL, before hepatectomy	≤100	2207	81.2	65.0	Reference	‐	Reference			
	>100	334	62.3	42.9	<.001	‐	1.879	1.280	2.758	.001
CEA, ng/mL, before hepatectomy	≤100	1530	83.0	66.5	Reference	‐	Reference			
	>100	1025	72.4	55.5	<.001	‐	1.376	0.815	2.322	.232
Characteristics of CRLM										
Number of CRLMs	1	1321	81.5	67.7	Reference	‐	Reference			
	2–4	977	77.5	60.0	<.001	Reference	1.484	1.014	2.174	.042
	5–	461	74.4	52.7	<.001	.014	1.142	0.690	1.891	.605
Maximum diameter of CRLM, cm	≤3	1845	80.6	64.6	Reference	‐	Reference			
	>3, ≤ 5	514	78.8	61.1	.236	Reference	1.001	0.668	1.499	.996
	>5	400	71.2	53.8	<.001	0.012	.898	0.570	1.413	.641
Distribution of CRLM	Unilobar	1813	81.0	66.4	Reference	‐	Reference			
	Bilobar	933	74.8	55.4	<.001	‐	1.022	0.709	1.474	.907
Initial resectability^d^	Resectable	2397	79.4	63.8	Reference	‐	Reference			
	Marginally resectable	179	73.4	56.4	.012	Reference	1.044	0.623	1.748	.871
	Initially unresectable	179	78.7	51.4	.004	0.743	0.969	0.567	1.658	.910
Operative findings										
Intraoperative red cell transfusion	No	2083	81.1	64.8	Reference	‐	Reference			
	Yes	517	71.3	53.5	<.001	‐	1.529	0.987	2.369	.057
Amount of intraoperative blood loss, g	≤500	1625	81.5	66.4	Reference	‐	Reference			
	>500, ≤1000	527	76.0	57.4	<.001	Reference	1.100	0.678	1.783	.700
	>1000	368	72.9	52.6	< 0.001	0.270	0.940	0.573	1.543	.807
Operation time, min	≤240	737	82.5	68.1	Reference	‐	Reference			
	>240, ≤480	1442	79.7	62.9	.043	Reference	0.920	0.639	1.325	.655
	>480	468	71.7	51.8	<.001	< 0.001	1.358	0.864	2.132	.184
Anatomical hepatectomy	No	1508	80.3	64.5	Reference	‐	NA			
	Yes	1216	77.1	60.2	.055	‐				
Laparoscopic surgery	Yes	685	82.6	66.9	Reference	‐	Reference			
	No	1938	77.8	61.1	.002	‐	1.192	0.838	1.697	0.329
Surgical curability^c^	R0	2462	79.9	63.9	Reference	‐	Reference			
	R1	183	71.7	53.6	.003	Reference	1.238	0.666	2.303	.500
	R2	43	48.1	24.2	<.001	< 0.001	2.593	1.246	5.396	.011
Surgical margin, mm	≥10	403	84.8	67.6	Reference		Reference			
	>0, <10	861	78.0	61.5	.042	Reference	1.033	0.705	1.512	.869
	0	355	72.5	56.1	<.001	0.041	1.018	0.632	1.639	.942
Pathological hilar lymph node metastasis	Negative	1965	78.7	61.4	Reference	‐	NA^e^			
	Positive	28	38.6	24.1	<.001	‐				
Postoperative findings										
Postoperative complication (Clavien‐Dindo classification ≥ III)	No	2430	80.4	63.8	Reference	‐	Reference			
	Yes	293	66.9	50.2	<.001	‐	1.384	0.855	2.242	.186
Postoperative chemotherapy^b^	Yes	468	84.0	68.4	Reference	‐	Reference			
	No	1464	78.7	60.9	.010	‐	1.286	0.795	2.082	.306

Abbreviations: CI, confidence interval; CRLM, colorectal liver metastasis; ly0, no lymphatic invasion; ly1, minimal lymphatic invasion; ly2, moderate lymphatic invasion; ly3, severe lymphatic invasion; NA, not applicable; N0, no evidence of lymph node metastasis; N1, metastasis in 1–3 pericolic/perirectal or intermediate lymph nodes; N2, metastasis in four or more pericolic/perirectal or intermediate lymph nodes; N3, metastasis in the main lymph node(s). In lower rectal cancer, metastasis in the main and/or lateral lymph node(s); R0, no residual tumor or no residual tumor with concomitant complete ablation therapy; R1, microscopic residual tumor; R2, macroscopic residual tumor; T1, tumor invades submucosa; T2, tumor invades muscularis propria; T3, tumor invades through the muscularis propria into the subserosa, or into nonperitonealized pericolic or perirectal tissues; T4, tumor directly invades other organs or structures, and/or perforates visceral peritoneum; tub1, well differentiated type tubular adenocarcinoma; tub2, moderately differentiated type tubular adenocarcinoma; v0, no venous invasion; v1, minimal venous invasion; v2, moderate venous invasion; v3, severe venous invasion; 3‐y, 3‐year survival rate; 5‐y, 5‐year survival rate.

^a^ Patients who underwent concomitant ablation therapy were excluded from the analyses.

^b^ The patients who underwent postoperative chemotherapy alone were included in the group of postoperative chemotherapy and those who underwent preoperative chemotherapy alone or both pre‐ and postoperative chemotherapy were included in the group of preoperative chemotherapy.

^c^ Described in accordance with General Rules for Clinical and Pathological Studies on Cancer of the Colon, Rectum and Anus (seventh edition, revised version, January 2009).

^d^ Resectable: All tumors could be removed with preservation of a negative surgical margin and sufficient remnant liver volume without performing two‐stage hepatectomy with portal vein embolization or major vessel reconstruction. Marginally resectable: All tumors could be removed with preservation of remnant liver volume, regardless of surgical margin, in two‐stage hepatectomy with portal vein embolization or major vessel reconstruction. Initially unresectable: All tumors could not be removed with preservation of sufficient remnant liver volume even if two‐stage hepatectomy with portal vein embolization or major vessel reconstruction was performed.

^e^ Excluded from multivariate analyses because of small numbers.

**TABLE 5:** Uni‐ and multivariate analyses for recurrence‐free survival after the hepatectomy alone for CRLM using the data of 2013–2017.

			Univariate analysis^a^				Multivariate analysis			
		*n*	3‐y	5‐y	*p*‐value	*p*‐value	Hazard ratio	95% CI		*p*‐value
Patient characteristics										
Age, years	<70	1273	35.9	31.4	Reference	‐	NA			
	≥70	1316	36.0	29.6	.689	‐				
Sex	Female	979	36.9	33.5	Reference	‐	NA			
	Male	1610	35.3	28.7	.103	‐				
Body mass index	≥25	522	37.9	32.4	Reference		NA			
	<25	2010	35.4	29.9	.140					
Preoperative MRI	Yes	1004	38.4	33.2	Reference	‐	NA			
	No	1543	34.5	28.8	.096	‐				
Preoperative chemotherapy^b^	No	912	41.7	35.8	Reference	‐	Reference			
	Yes	925	26.5	22.2	<.001	‐	1.171	.923	1.484	.193
Emergence time of CRLM	Metachronous	1185	44.6	38.6	Reference	‐	Reference			
	Synchronous	1404	28.7	23.8	<.001	‐	1.268	1.047	1.536	.015
Concomitant extrahepatic metastasis	No	2286	38.4	32.6	Reference	‐	Reference			
	Yes	294	17.2	14.9	<.001	‐	1.431	1.123	1.823	.004
Characteristics of primary lesion										
Location of primary lesion	Left side	1853	36.1	30.4	Reference	‐	NA			
	Right side	688	35.6	30.8	.873	‐				
Pathological tumor depth of primary lesion^c^	T1/T2	226	50.0	42.5	Reference	‐	Reference			
	T3/T4	2275	34.5	29.1	<.001	‐	1.268	0.936	1.717	.126
Lymphatic invasion^c^	ly0/1	1932	37.8	32.0	Reference	‐	Reference			
	ly2/3	574	29.4	25.1	<.001	‐	1.308	1.057	1.619	.014
Venous invasion^c^	v0/1	1363	38.4	33.0	Reference	‐	Reference			
	v2/3	1126	32.4	27.4	<.001	‐	1.021	0.858	1.215	.815
Lymph node metastasis^c^	N0	851	48.3	40.2	Reference	‐	Reference			
	N1	1027	32.6	28.3	<.001	Reference	1.421	1.171	1.724	<.001
	N2/3	612	24.2	20.8	<.001	< 0.001	1.435	1.137	1.810	.002
Histological type^c^	tub1	670	42.7	36.7	Reference	‐	Reference			
	tub2	1721	33.5	28.4	<.001	Reference	1.053	0.694	1.596	.809
	others	139	33.6	27.8	.005	0.588	1.355	0.915	2.005	.129
KRAS status	Wild	823	27.4	22.8	Reference	‐	NA			
	Mutant	534	25.5	22.3	.073	‐				
Tumor marker value										
CA19‐9, U/mL, before hepatectomy	≤100	2088	38.5	32.6	Reference	‐	Reference			
	>100	309	19.0	16.1	<.001	‐	1.752	1.374	2.233	<.001
CEA, ng/mL, before hepatectomy	≤100	1444	40.7	35.1	Reference	‐	Reference			
	>100	967	29.1	23.7	<.001	‐	1.060	.882	1.274	.534
Characteristics of CRLM										
Number of CRLMs	1	1266	45.8	39.9	Reference	‐	Reference			
	2–4	910	29.5	24.3	<.001	Reference	1.224	.982	1.526	.072
	5–	413	20.1	15.1	<.001	< 0.001	1.376	1.017	1.862	.039
Maximum diameter of CRLM, cm	≤3	1748	38.9	33.4	Reference	‐	Reference			
	>3, ≤5	483	34.5	29.7	.053	Reference	0.949	.761	1.183	.641
	>5	358	22.9	17.0	<.001	< 0.001	1.019	.788	1.318	.888
Distribution of CRLM	Unilobar	1718	41.3	35.6	Reference	‐	Reference			
	Bilobar	862	25.3	20.2	<.001	‐	1.090	.870	1.364	.454
Initial resectability	Resectable	2286	37.9	32.3	Reference	‐	Reference			
	Marginally resectable	157	22.4	16.9	<.001	Reference	1.071	.781	1.470	.671
	Initially unresectable	142	19.5	17.6	<.001	0.776	.868	0.598	1.259	.456
Operative findings										
Intraoperative red cell transfusion	No	1963	37.2	31.4	Reference	‐	Reference			
	Yes	483	29.6	25.1	<.001	‐	1.062	0.792	1.422	.689
Amount of intraoperative blood loss, g	≤ 500	1533	40.1	34.5	Reference	‐	Reference			
	>500, ≤1000	494	28.2	23.3	<.001	Reference	1.225	.995	1.507	.056
	>1000	340	27.2	22.8	<.001	0.812	0.840	.616	1.145	.270
Operation time, min	≤240	699	44.5	38.7	Reference	‐	Reference			
	>240, ≤480	1364	34.9	29.3	<.001	Reference	1.223	.992	1.509	.060
	>480	422	25.0	21.0	<.001	< 0.001	1.314	.995	1.737	.055
Anatomical hepatectomy	No	1422	38.2	32.1	Reference	‐	Reference			
	Yes	1134	33.4	28.9	.019	‐	1.035	.873	1.226	.695
Laparoscopic surgery	Yes	653	41.8	35.9	Reference	‐	Reference			
	No	1814	34.0	28.6	<.001	‐	1.243	1.010	1.529	.040
Surgical curability^c^ ^,^ ^d^	R0	2354	36.8	31.2	Reference	‐	Reference			
	R1	175	25.4	21.7	<.001	Reference	1.357	.937	1.965	.106
Surgical margin, mm	≥10	384	42.2	36.6	Reference	‐	Reference			
	>0, <10	815	35.1	30.6	.042	Reference	1.096	.888	1.353	.392
	0	333	26.2	20.8	<.001	< 0.001	1.153	.873	1.522	.316
Pathological hilar lymph node metastasis	Negative	1841	35.2	29.4	Reference	‐	NA^e^			
	Positive	26	19.0	9.5	.008	‐				
Postoperative findings										
Postoperative complication (Clavien‐Dindo classification ≥ III)	No	2291	36.9	31.4	Reference	‐	Reference			
	Yes	266	28.3	23.6	<.001	‐	1.219	.942	1.576	.132
Postoperative chemotherapy†	Yes	448	40.6	34.7	Reference	‐				
	No	1358	32.0	27.0	<.001	‐	1.240	.973	1.579	.082

*Note*: Resectable: All tumors could be removed with preservation of a negative surgical margin and sufficient remnant liver volume without performing two‐stage hepatectomy with portal vein embolization or major vessel reconstruction. Marginally resectable: All tumors could be removed with preservation of remnant liver volume, regardless of surgical margin, in two‐stage hepatectomy with portal vein embolization or major vessel reconstruction. Initially unresectable: All tumors could not be removed with preservation of sufficient remnant liver volume even if two‐stage hepatectomy with portal vein embolization or major vessel reconstruction was performed.

Abbreviations: CI, confidence interval; CRLM, colorectal liver metastasis; ly0, no lymphatic invasion; ly1, minimal lymphatic invasion; ly2, moderate lymphatic invasion; ly3, severe lymphatic invasion; MRI, magnetic resonance imaging; NA, not applicable; N0, no evidence of lymph node metastasis; N1, metastasis in 1–3 pericolic/perirectal or intermediate lymph nodes; N2, metastasis in four or more pericolic/perirectal or intermediate lymph nodes; N3, metastasis in the main lymph node(s). In lower rectal cancer, metastasis in the main and/or lateral lymph node(s); R0, no residual tumor or no residual tumor with concomitant complete ablation therapy; R1, microscopic residual tumor; R2, macroscopic residual tumor; T1, tumor invades submucosa; T2, tumor invades muscularis propria; T3, tumor invades through the muscularis propria into the subserosa, or into nonperitonealized pericolic or perirectal tissues; T4, tumor directly invades other organs or structures, and/or perforates visceral peritoneum; tub1, well differentiated type tubular adenocarcinoma; tub2, moderately differentiated type tubular adenocarcinoma; v0, no venous invasion; v1, minimal venous invasion; v2, moderate venous invasion; v3, severe venous invasion; 3‐y, 3‐year survival rate; 5‐y, 5‐year survival rate.

^a^ Patients who underwent concomitant ablation therapy were excluded from the analyses.

^b^ The patients who underwent postoperative chemotherapy alone were included in the group of postoperative chemotherapy and those who underwent preoperative chemotherapy alone or both pre‐ and postoperative chemotherapy were included in the group of preoperative chemotherapy.

^c^ Described in accordance with General Rules for Clinical and Pathological Studies on Cancer of the Colon, Rectum and Anus (seventh edition, revised version, January 2009).

^d^ The patients with R2 resection were excluded from the analyses.

^e^ Excluded from multivariate analyses because of small numbers.

## 4 DISCUSSION

The present study included data registered in a comprehensive nationwide database by institutions certified by the JSCCR and JSHBPS. Accordingly, all patients were treated by colorectal cancer specialists and board‐certified hepatic surgeons, which ensured the quality and strategies of treatment. In summary, compared to the 2005–2007 data, the age at diagnosis of CRLM was higher in the 2013–2017 data, which might be due to societal aging. Regarding the CRLM characteristics, although the number of CRLM was higher in the 2013–2017 data, maximum CRLM tumor diameter at diagnosis was smaller in the 2013–2017 data. Several factors, such as advances in imaging modalities or more aggressive surgical approaches due to improvements in surgical outcomes, might have affected these results.^2^ The higher rate of laparoscopic surgery in recent studies may have led to longer operative times and lesser blood losses. Furthermore, advances in surgical procedures and perioperative treatment might have led to lower 90‐day mortality in these years. Analyzing the data from 2013 to 2017 compared to the prognostic data from 2005 to 2007, it is noteworthy that even though patients who underwent hepatectomy in the later period exhibited a higher burden of prognostic risk factors, including elevated CRLM numbers, synchronous metastasis and higher JSHBPS nomogram score, their OS was demonstrably improved (*p* < .001). In contrast, their RFS did not show a statistically significant difference (*p* = .068). Although RFS was affected by higher JSHBPS nomogram score in the data from 2013–2017, advance of treatment after recurrence might contribute to improve OS in the 2013–2017 data than the 2005–2007 data. The lower proportion of the patients who underwent adjuvant chemotherapy after hepatectomy in 2013–2017 group (50.7% vs. 67.2%, *p* < .001; Table S6) might have impact for the results of RFS in both groups as well. Furthermore, multidisciplinary treatment including infusional fluorouracil, leucovorin, and oxaliplatin/irinotecan (FOLFOX/FOLFIRI) therapy or molecular targeting agents might contribute to improve OS in the 2013–2017 data than the 2005–2007 data. However, no evidence was shown in the present study and further precise study should be warranted. In addition, the definitive reason for the disparities of OS and RFS is unclear, but differences in the follow‐up periods might have affected the results. Although RFS had reportedly strong correlation with OS in CRLM,^9^ several reports showed the discrepancy between RFS and OS.^14^, ^15^, ^16^, ^17^

This study identified various independent prognostic predictors in patients who underwent hepatectomy for CRLM. However, regarding perioperative chemotherapy, both pre−/postoperative chemotherapy had significant impact for prognoses in the univariate analyses but none was selected as an independent prognosticator in multivariate analyses. The patients underwent preoperative chemotherapy showed poorer survival than those with no preoperative chemotherapy in both OS and RFS. However, since the definition of neoadjuvant, induction chemotherapy, or conversion chemotherapy was not unified in the present study, it was difficult to show clear evidence in the present study. Regarding postoperative chemotherapy, although the RFS was reportedly shown to be improved by adjuvant chemotherapy after hepatectomy for CRLM.^16^, ^18^, ^19^, ^20^ However, its impact for OS is inconsistent.^16^, ^18^, ^19^, ^20^ The patients who underwent postoperative chemotherapy showed better survival in both OS and RFS in the present study only in univariate analysis. Nevertheless, since the data regarding perioperative chemotherapy showed some lacks in the present data, enough evidence was difficult to show.

Concomitant extrahepatic metastasis was selected as an independent predictor of RFS. Our previous study using data from 2005 to 2007 reported that the prognosis is more favorable for controllable lung metastasis than for distant lymph node metastasis or peritoneal dissemination.^7^ More detailed stratification of extrahepatic metastasis is required to select good candidates for resection of CRLM in patients with concomitant extrahepatic metastasis.

The present multivariate analysis selected lymph node metastases as an independent prognostic factor associated with the primary lesion. Many previous studies reported similar results.^14^, ^17^, ^21^, ^22^, ^23^, ^24^, ^25^, ^26^, ^27^, ^28^, ^29^ Although the present study highlights the importance of pathological findings of the primary lesion, its curability was not considered. Studies addressing the curability of primary lesions for the prognosis of CRLM should be considered.

Regarding preoperative tumor marker values, the present study identified CA19‐9 levels >100 U/mL as an independent predictor of prognosis, whereas CEA >100 ng/mL were not considered. Hazard ratios (HRs) of CA19‐9 >100 U/mL in the present study were higher for both OS and RFS than for other factors (1.698 and 1.476, respectively). Several studies have also shown a greater hazard ratio for CA19‐9 than for CEA as a prognostic predictor in resectable CRLM.^21^, ^29^, ^30^, ^31^ Although previous studies have reported that high CEA values are strong independent predictors of patient survival,^22^, ^23^, ^24^, ^25^, ^26^, ^27^, ^28^ these studies did not evaluate CA19‐9 values. However, because the cutoff values varied among studies, further studies are needed to evaluate the usefulness of tumor markers in clinical practice.

Regarding the characteristics of CRLM, the number of CRLM was identified as an independent predictor of poor prognosis, whereas the CRLM diameter was not selected. Previous reports have shown that the hazard ratio for long‐term prognosis is greater in tumors than in tumors.^8^, ^11^, ^32^ We previously published a novel H classification that focused more on tumor number than tumor size using data from 2005 to 2007.^8^ Number of CRLM ≥5, which is high risk factor of recurrences, should be considered carefully for the indication of hepatectomy,^32^, ^33^, ^34^, ^35^ and was selected as independent predictor of RFS in the present study. Not only for tumor recurrences, CRLM number ≥5 showed higher hazard ratio for OS than CRLM number 2–4 in the previous study (hazard ratio 2.69 and 1.51 with reference to solitary CRLM) which enrolled the patients in 2000–2004.^14^ However, the number of CRLM ≥5 was not selected as independent predictor of OS in the present study and its reason was unclear.

R2 resection was found to be the strongest independent predictor of OS. However, the surgical curability of R1 was not selected as an independent prognostic factor for either OS or RFS in patients who underwent hepatectomy for CRLM. Recently, for patients who underwent preoperative chemotherapy, several studies have shown a positive prognostic impact of hepatectomy, even in those who underwent R1 resection.^36^, ^37^ Recent studies evaluated the prognostic impact of various types of R1, such as tumor exposure in adjacent major vessels.^38^ Further studies are necessary to more precisely evaluate the relevance of R1.

Nonlaparoscopic approach was selected as independent predictor of poor RFS in the present study. Laparoscopic approach was significantly increased in the 2013–2017 data than that from the 2005–2007 data (Table 3). Although the patient selection criteria for laparoscopic surgery might have impact for RFS in the present study, no evidence was shown. Further precise study such as evaluating the difficulty of the laparoscopic procedure is needed.

A major limitation of this study was the single ethnicity of the patients. Furthermore, this study did not cover all the data of patients with CRLM in Japan. In addition, data were lacking because of the retrospective study design. As the database used in the present study was created in 2014, the latest important information, such as *BRAF* status, was not collected.^2^, ^39^, ^40^, ^41^ Therefore, we are attempting to create a new database system. However, we believe that this study will be of use to physicians wishing to use our nationwide database to conduct meaningful studies for further development of treatments for CRLM and will also be of value to physicians involved in CRLM treatment. In conclusion, analyses conducted using a nationwide database system of patients diagnosed with CRLM revealed a transition in the characteristics of CRLM and various prognosticators in patients who underwent hepatectomy. The OS in the 2013–2017 data was better than that in the 2005–2007 data, although patients with more advanced CRLM were included.

## CONFLICT OF INTEREST STATEMENT

None of the authors has any conflicts of interest to disclose regarding this study.

## Supporting information


Data S1.

Data S2.

Data S3.

Data S4.

Data S5.

Data S6.

Data S7.

Data S8.

Data S9.
