# Supplementary figures and images for: Comprehensive data of 5085 patients newly diagnosed with colorectal liver metastasis between 2013 and 2017: Fourth report of a nationwide survey in Japan
Source: J Hepatobiliary Pancreat Sci. 2024 Nov 12;32(1):26–43. doi: 10.1002/jhbp.12078 (PMC11780304; doi:10.1002/jhbp.12078)

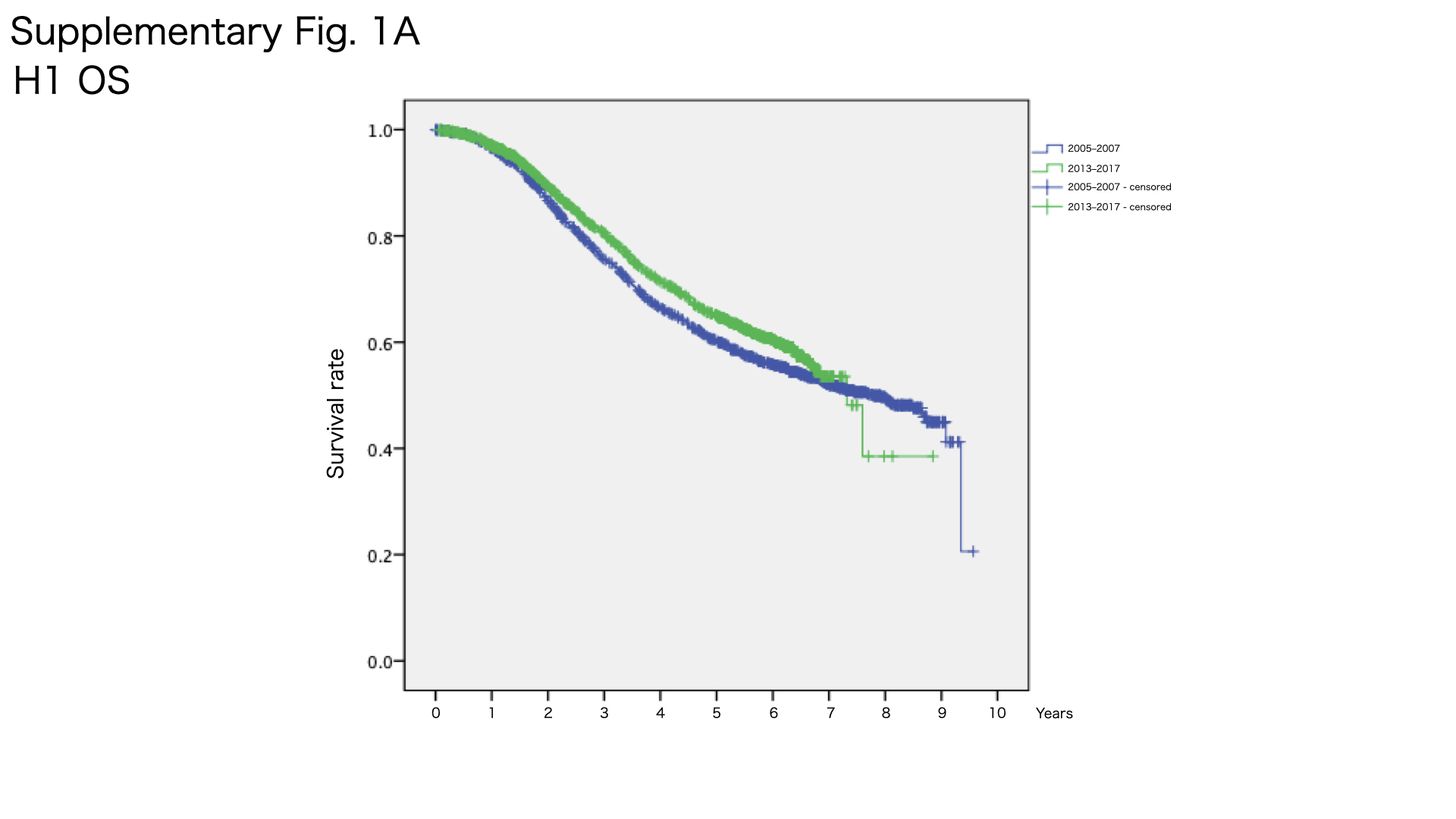

Supplement: Supplementary file 1 — Data S1. [file JHBP-32-26-s007.jpeg]

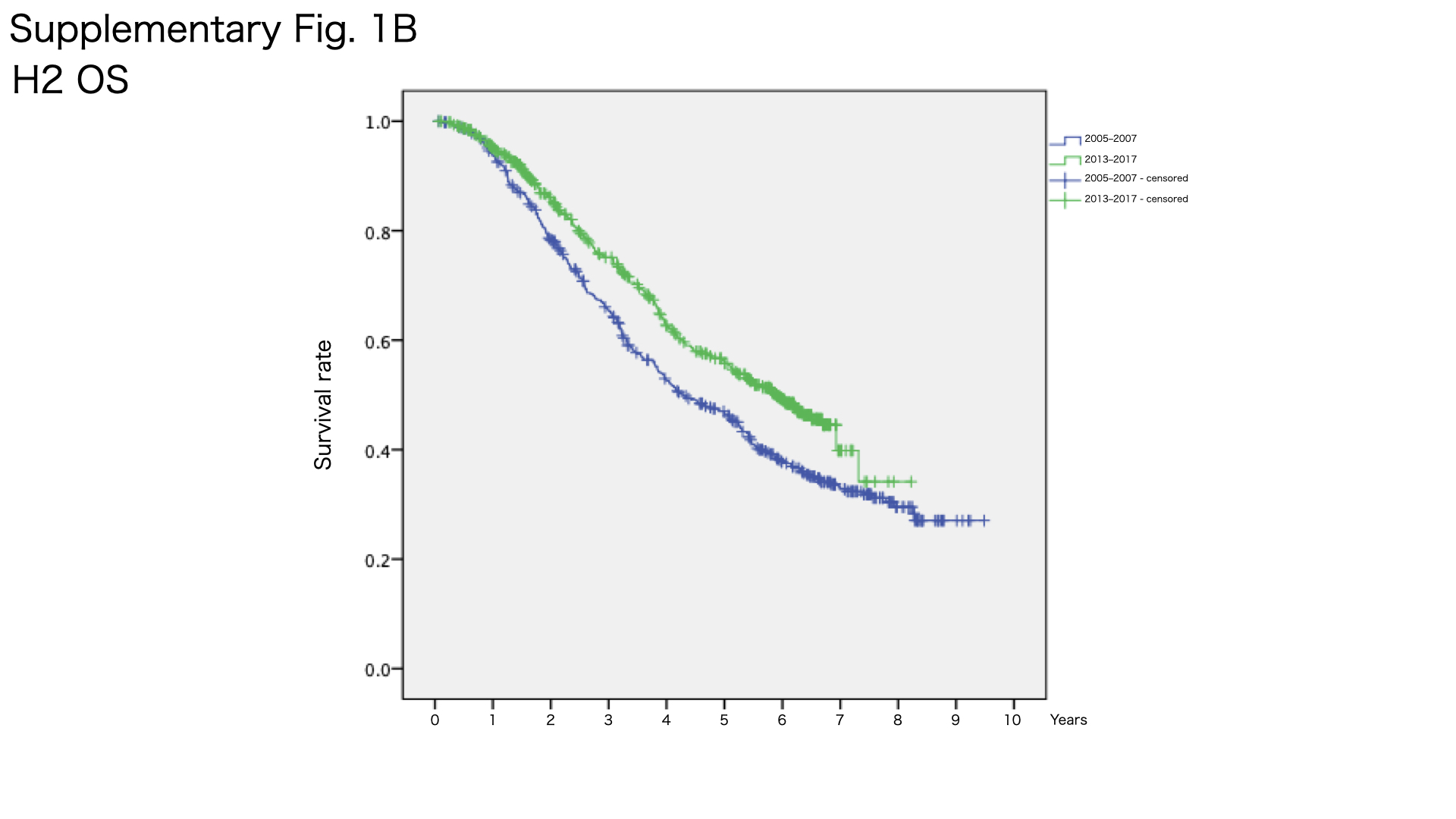

Supplement: Supplementary file 2 — Data S2. [file JHBP-32-26-s008.jpeg]

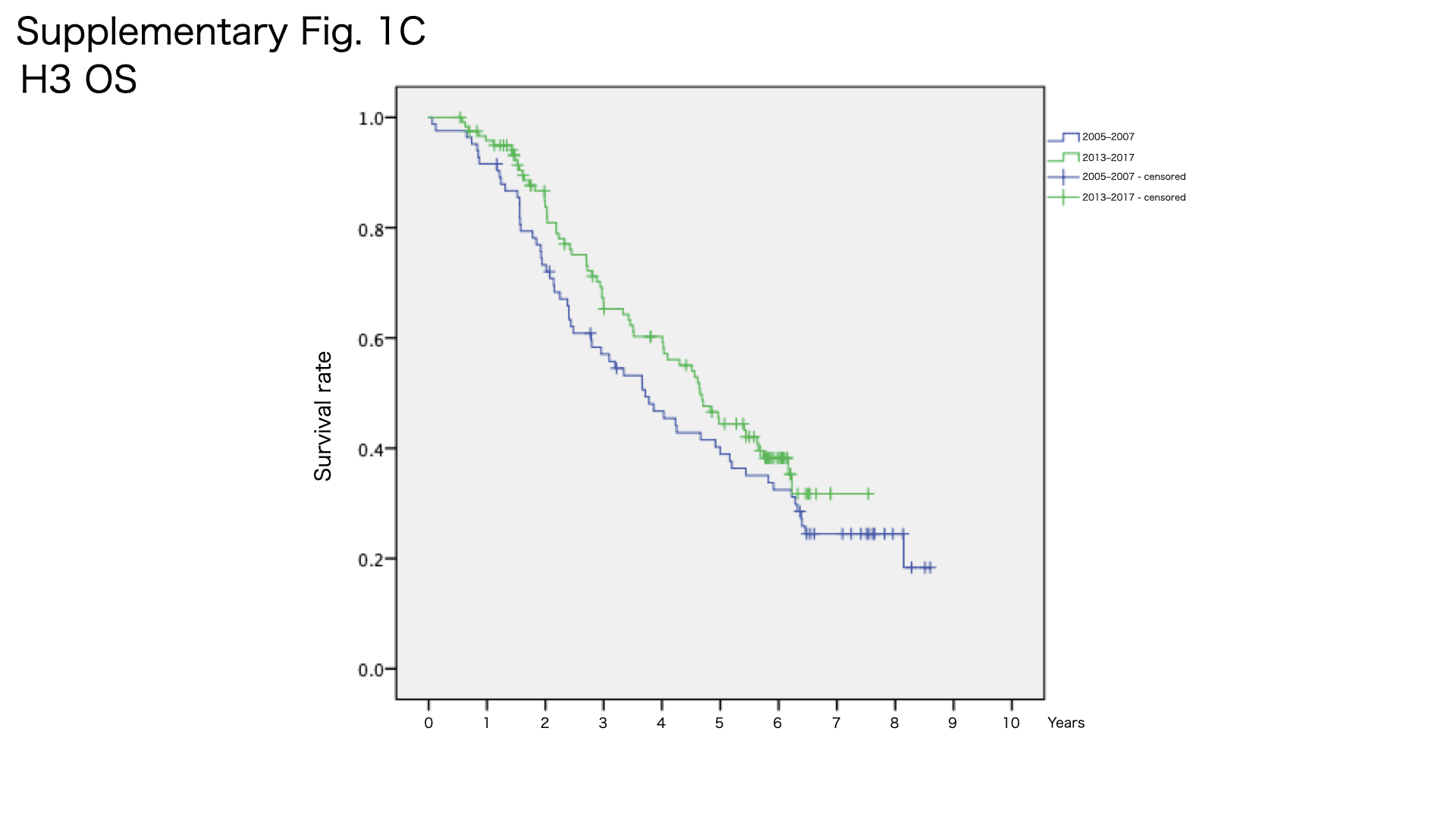

Supplement: Supplementary file 3 — Data S3. [file JHBP-32-26-s002.jpeg]

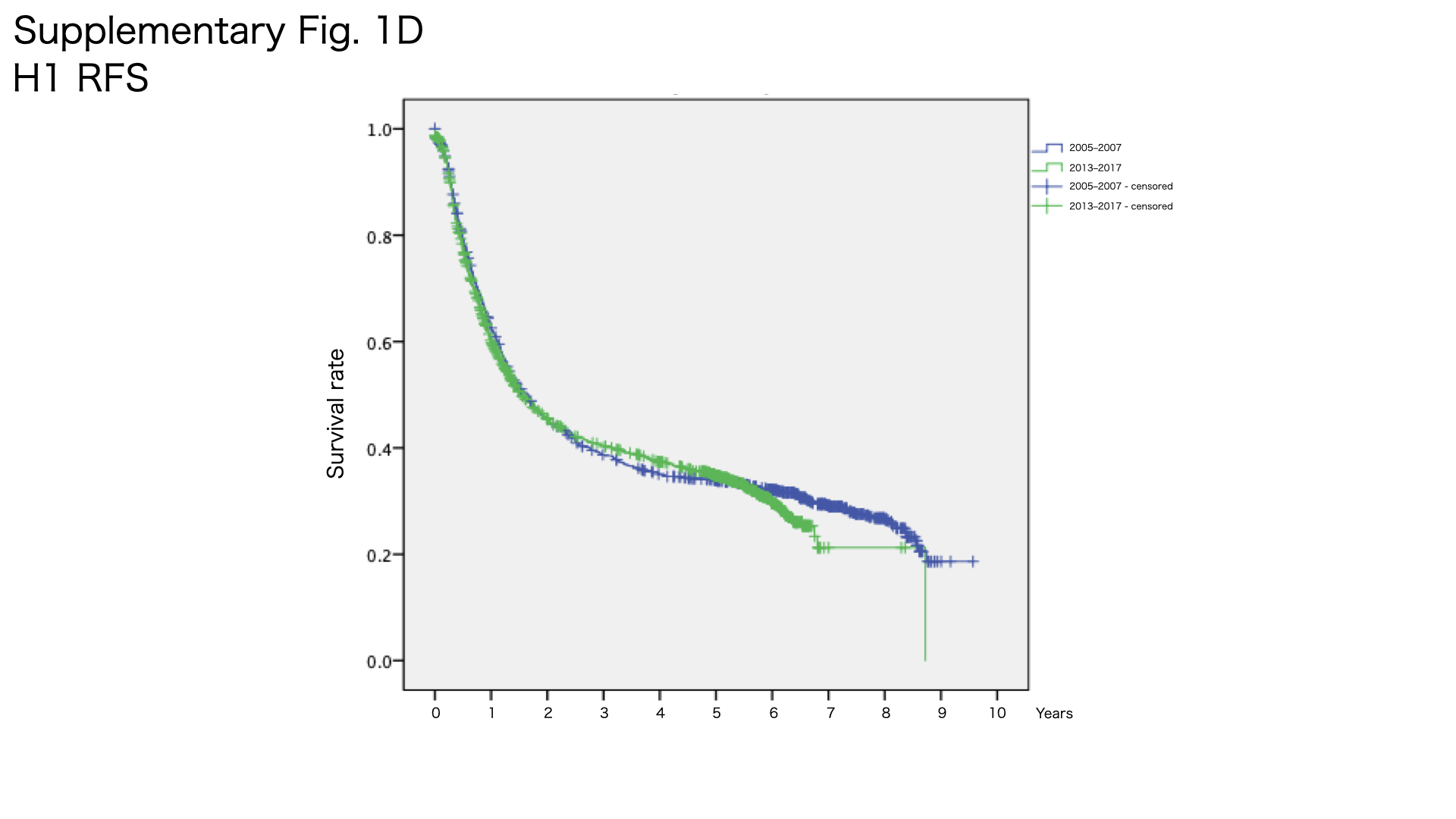

Supplement: Supplementary file 4 — Data S4. [file JHBP-32-26-s001.jpeg]

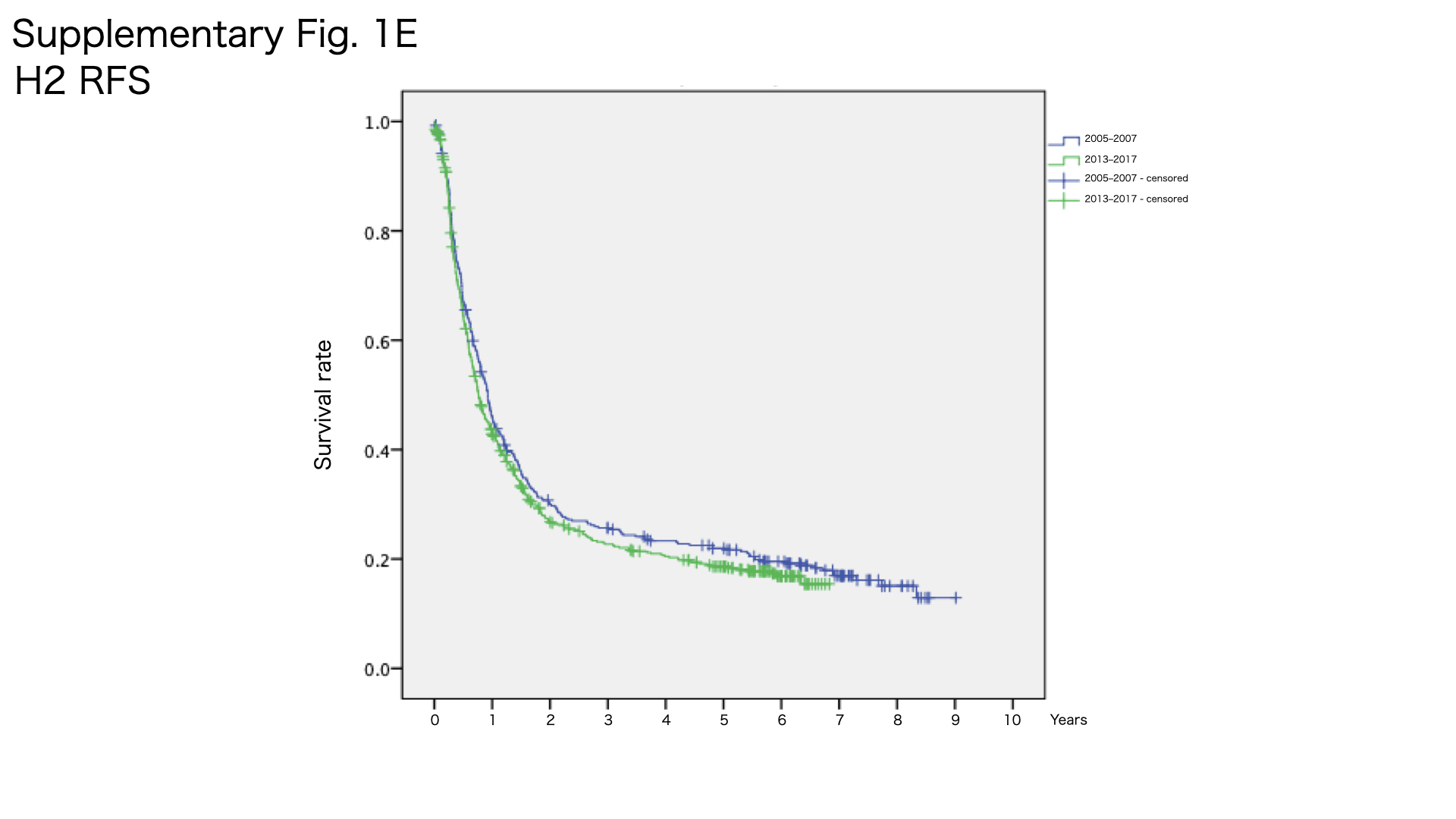

Supplement: Supplementary file 5 — Data S5. [file JHBP-32-26-s003.jpeg]

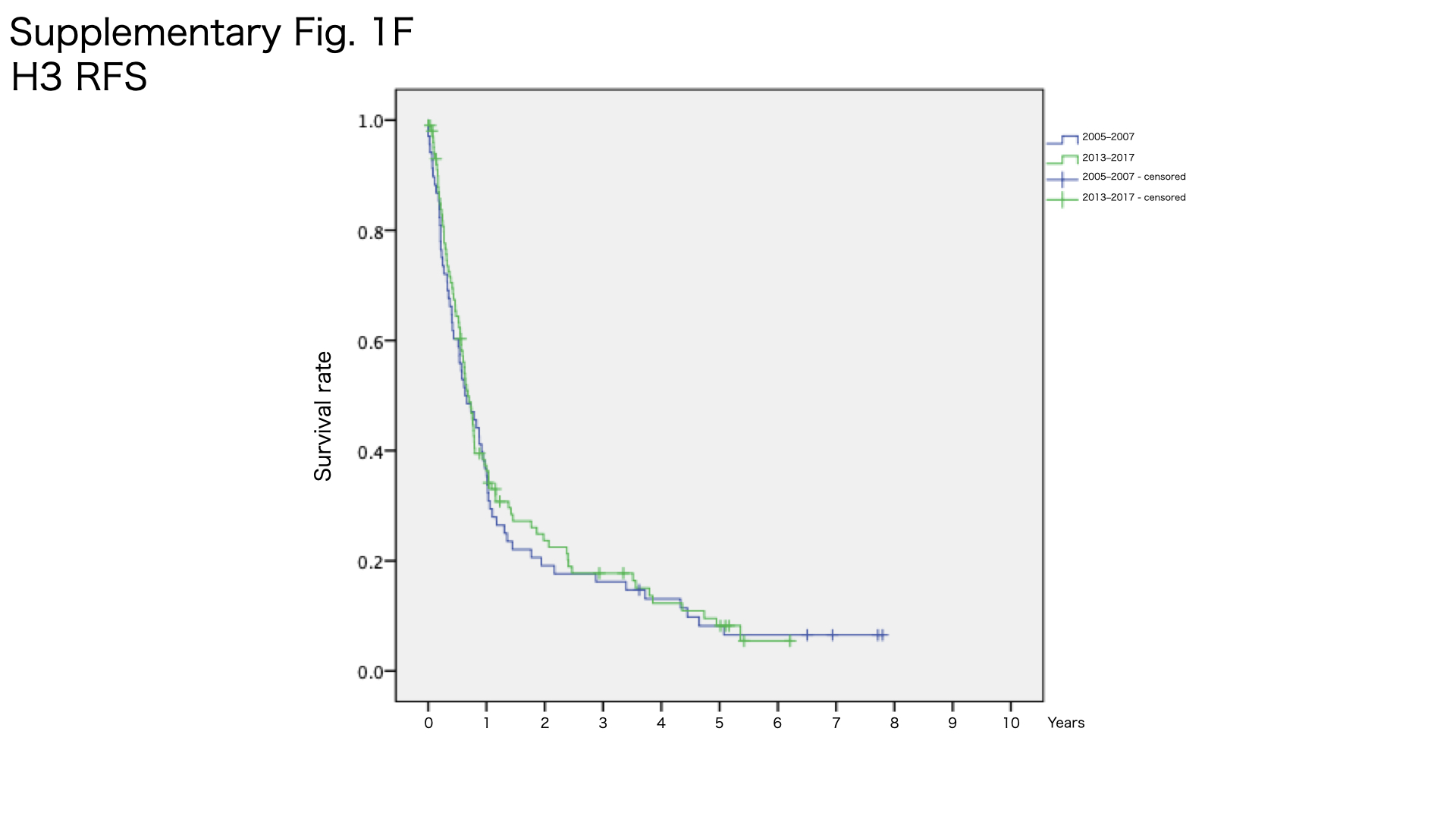

Supplement: Supplementary file 6 — Data S6. [file JHBP-32-26-s004.jpeg]

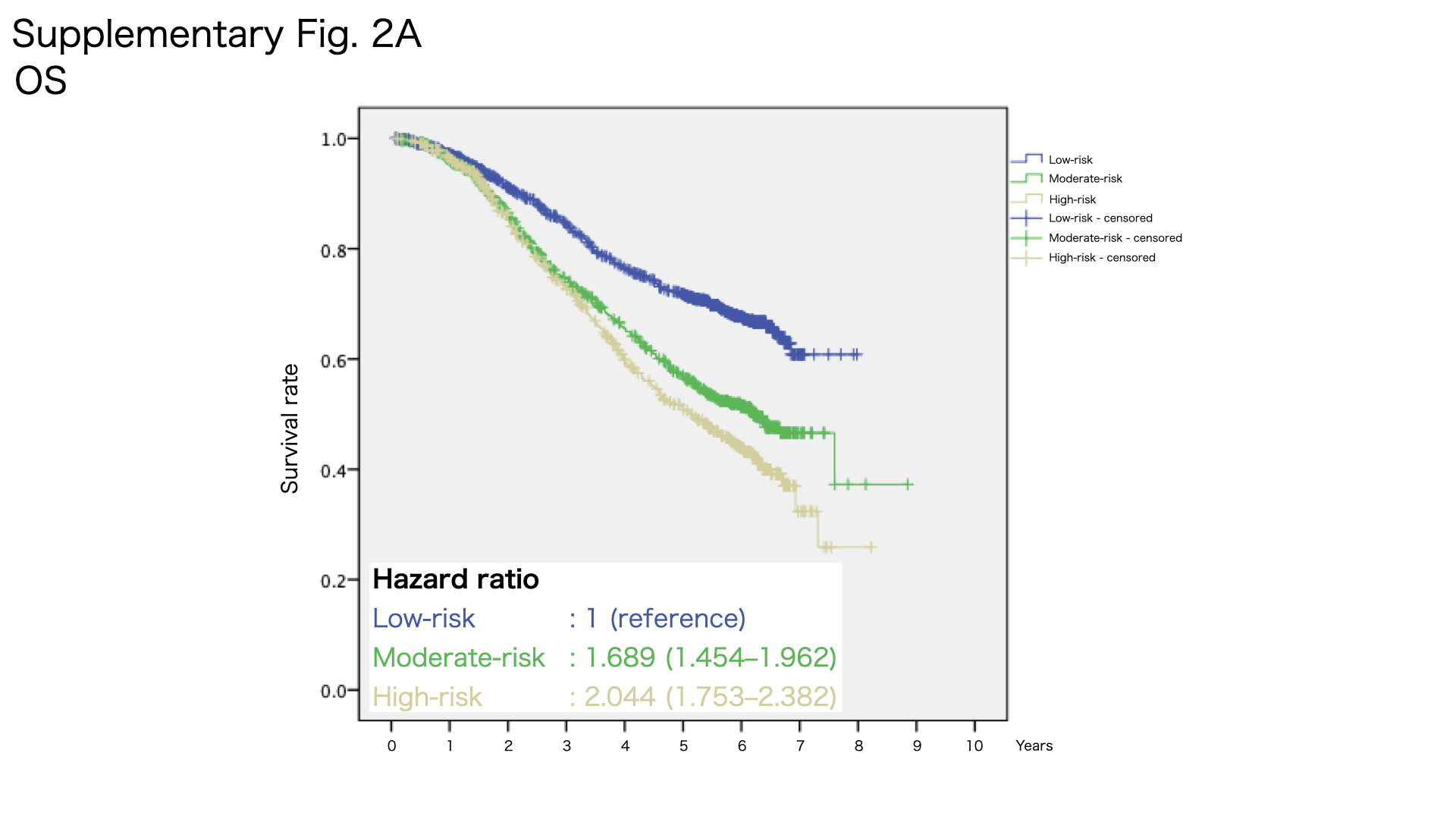

Supplement: Supplementary file 7 — Data S7. [file JHBP-32-26-s005.jpeg]

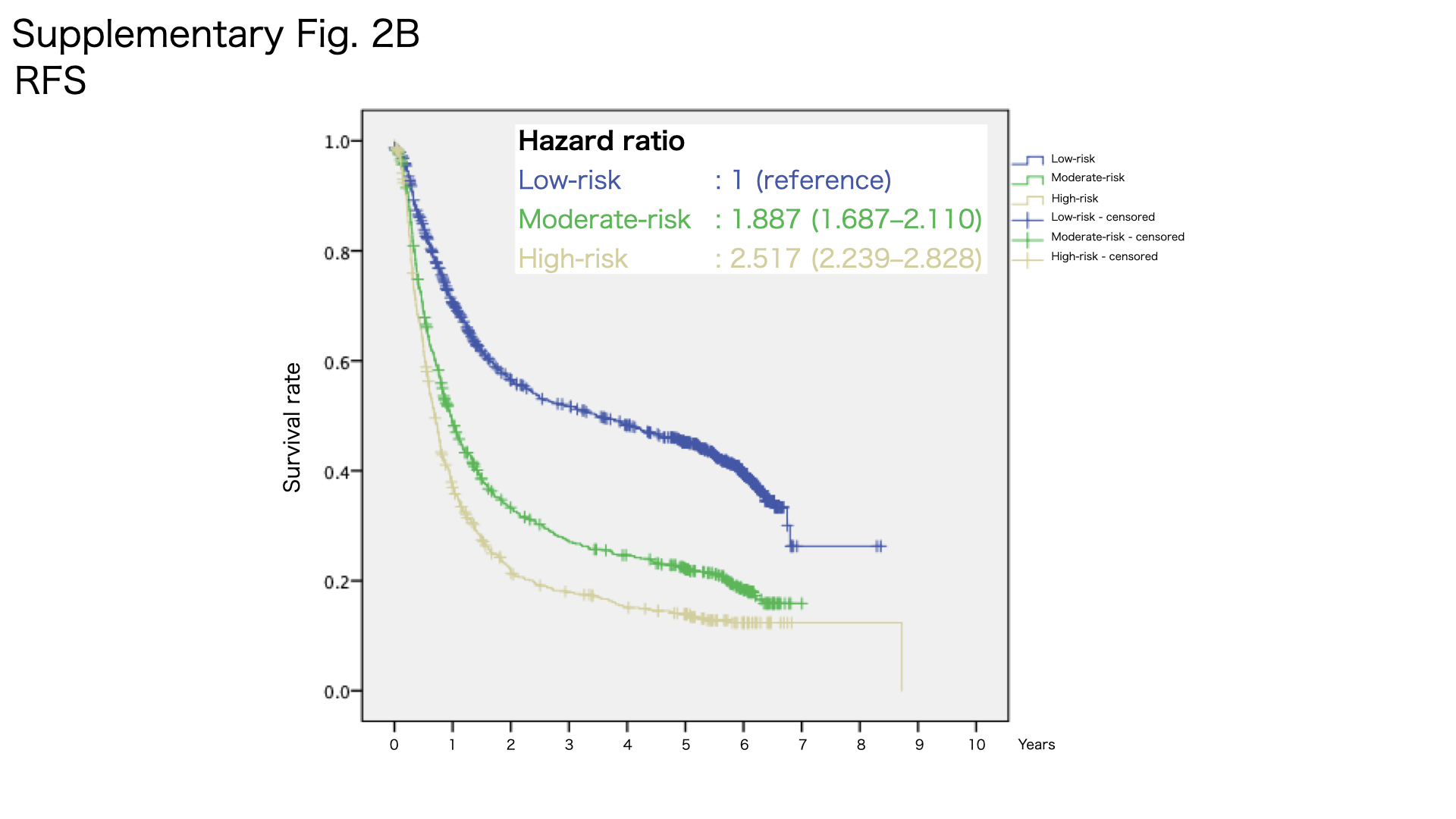

Supplement: Supplementary file 8 — Data S8. [file JHBP-32-26-s006.jpeg]
