# Supplementary material for: Comprehensive data of 5085 patients newly diagnosed with colorectal liver metastasis between 2013 and 2017: Fourth report of a nationwide survey in Japan
Source: J Hepatobiliary Pancreat Sci. 2024 Nov 12;32(1):26–43. doi: 10.1002/jhbp.12078 (PMC11780304; doi:10.1002/jhbp.12078)
Supplement: Supplementary file 9 — Data S9. [file JHBP-32-26-s009.docx]

**Table S1-1** Patients’ characteristics (all patients with CRLM)

|  | | 2013–2017 | 2005–2007 | *P* value |
| --- | --- | --- | --- | --- |
|  |  | ***n* = 5,085** | ***n* = 3,820** |  |
| Age, y | Median (25, 75%) | 67 (60, 75) | 65 (57, 73) | < 0.001 |
| Sex | Male | 3,144 (61.8%) | 2,364 (61.9%) | 0.957 |
| Height, cm | Median (25, 75%) | 161.0 (154.0, 167.4) | 160.0 (153.0, 167.0) | 0.002 |
|  | Missing | 155 | 754 |  |
| Weight, kg | Median (25, 75%) | 56.5 (48.6, 65.0) | 56.0 (48.8, 63.9) | 0.049 |
|  | Missing | 159 | 751 |  |
| BMI | Median (25, 75%) | 21.80 (19.49, 24.17) | 21.89 (19.68, 24.01) | 0.803 |
|  | Missing | 168 | 770 |  |
| HBs-Ag | Positive | 92 (1.9%) | 59 (1.9%) | 0.920 |
|  | Missing | 366 | 743 |  |
| HCV-Ab | Positive | 144 (3.1%) | 111 (3.6%) | 0.201 |
|  | Missing | 377 | 723 |  |
| Emergence time of CRLM* | Synchronous | 3,364 (66.2%) | 2,410 (63.1%) | 0.003 |
|  | Metachronous | 17,21 (33.8%) | 1,410 (36.9%) |  |
| Resection of primary lesion | Yes | 4,365 (85.8%) | 3,500 (91.6%) | < 0.001 |
| Local treatment for CRLM** | Hepatectomy alone | 2,759 (54.3%) | 2,163 (56.6%) | < 0.001 |
|  | Hepatectomy + ablation | 29 (0.6%） | 62 (1.6%) |  |
|  | Ablation alone | 37 (0.7%) | 63 (1.6%) |  |

* Synchronous liver metastasis was defined as a metastatic liver tumor diagnosed in the period of preoperative examination or surgery for primary lesion

** Local treatment for CRLM included hepatectomy and ablation therapy (radiofrequency ablation and microwave coagulation therapy), but did not include hepatic arterial infusion chemotherapy

CRLM, colorectal liver metastasis; BMI, Body mass index; HBs-Ag, Hepatitis B virus surface antigen; HCV-Ab, Hepatitis C virus antibody

**Table S1-2** Patients’ characteristics (patients who underwent ablation therapy alone as local treatment for CRLM)

|  | | 2013–2017 | 2005–2007 | *P* value |
| --- | --- | --- | --- | --- |
|  |  | ***n* = 37** | ***n* = 63** |  |
| Age, y | Median (25, 75%) | 75 (66, 83) | 65 (60, 75) | 0.012 |
| Sex | Male | 23 (62.2%) | 45 (71.9%) | 0.338 |
| Height, cm | Median (25, 75%) | 156.8 (151.0, 162.4) | 161.6 (157.9, 166.0) | 0.015 |
|  | Missing | 1 | 13 |  |
| Weight, kg | Median (25, 75%) | 53.0 (45.8, 62.2) | 56.0 (50.2, 61.0) | 0.231 |
|  | Missing | 1 | 12 |  |
| BMI | Median (25, 75%) | 21.79 (19.30, 23.94) | 21.63 (19.55, 23.50) | 0.823 |
|  | Missing | 1 | 13 |  |
| HBs-Ag | Positive | 0 (0%) | 0 (0%) | - |
|  | Missing | 2 | 14 |  |
| HCV-Ab | Positive | 1 (2.9%) | 0 (0%) | 0.407 |
|  | Missing | 2 | 12 |  |
| Emergence time of CRLM* | Synchronous | 15 (40.5%) | 35 (55.6%) | 0.147 |
|  | Metachronous | 22 (59.5%) | 28 (44.4%) |  |
| Resection of primary lesion | Yes | 36 (97.3%) | 61 (96.8%) | 0.692 |

* Synchronous liver metastasis was defined as a metastatic liver tumor diagnosed in the period of preoperative examination or surgery for primary lesion

CRLM, colorectal liver metastasis; BMI, body mass index; HBs-Ag, hepatitis B virus surface antigen; HCV-Ab, hepatitis C virus antibody

**Table S2-1** Clinical findings of CRLM at diagnosis (all patients with CRLM)

|  |  | 2013–2017 | 2005–2007 | *P* value |
| --- | --- | --- | --- | --- |
|  |  | ***n* = 5,085** | ***n* = 3,820** |  |
| Number of CRLMs | Median (25, 75%) | 3 (1, 10) | 2 (1, 8) | 0.009 |
| Maximum diameter of CRLM, mm | Median (25, 75%) | 28 (17, 50) | 30 (19, 50) | 0.010 |
| Distribution of CRLMs | Bilobar | 2,412 (49.4%) | 1,758 (47.9%) | 0.168 |
|  | Unilobar | 2,470 (50.6%) | 1,911 (52.1%) |  |
|  | Missing | 203 | 152 |  |
| Local treatability on clinical findings* | Treatable | 2,626 (52.0%) | 2,117 (57.7%) | < 0.001 |
|  | Marginally treatable | 294 (5.8%) | 196 (5.3%) |  |
|  | Initially untreatable | 2,130 (42.2%) | 1,356 (37.0%) |  |
|  | Missing | 35 | 1.9 |  |
| Concomitant extrahepatic metastasis | Yes | 1,783 (35.3%) | 1,158 (31.2%) | < 0.001 |
|  | Missing | 39 | 108 |  |

- Treatable: All tumors could be removed with preservation of a negative surgical margin and sufficient remnant liver volume without performing two-stage hepatectomy with portal vein embolization or major vessel reconstruction. Marginally treatable: All tumors could be removed with preservation of remnant liver volume, regardless of surgical margin, in two-stage hepatectomy with portal vein embolization or major vessel reconstruction. Untreatable: All tumors could not be removed with preservation of sufficient remnant liver volume even if two-stage hepatectomy with portal vein embolization or major vessel reconstruction was performed. Lesions that could be completely ablated were included in the “treatable” category.

CRLM, colorectal liver metastasis

**Table S2-2** Clinical findings of CRLM at diagnosis (patients who underwent ablation therapy alone as local treatment for CRLM)

|  | | 2013–2017 | 2005–2007 | *P* value |
| --- | --- | --- | --- | --- |
|  |  | ***n* = 37** | ***n* = 63** |  |
| Number of CRLMs | Median (25, 75%) | 1 (1, 2) | 2 (1, 3) | 0.035 |
| Maximum diameter of CRLM, mm | Median (25, 75%) | 18 (14, 29) | 25 (15, 35) | 0.223 |
| Distribution of CRLMs | Bilobar | 9 (24.3%) | 18 (30.0%) | 0.545 |
|  | Unilobar | 28 (75.7%) | 42.0 (70.0%) |  |
|  | Missing | 0 | 3 |  |
| Local treatability on clinical findings* | Treatable | 26 (70.3%) | 52 (82.5%) | 0.052 |
|  | Marginally treatable | 1 (2.7%) | 5 (7.9%) |  |
|  | Initially untreatable | 10 (27.0%) | 6 (9.5%) |  |
| Concomitant extrahepatic metastasis | Yes | 12 (33.3%) | 11 (17.5%) | 0.072 |
|  | Missing | 1 | 0 |  |
| Pretreatment albumin, g/dL | Median (25, 75) | 4.0 (3.5, 4.2) | 4.1 (3.3, 4.3) | 0.674 |
|  | Missing | 3 | 11 |  |
| Pretreatment T-Bil, mg/dL | Median (25, 75) | 0.6 (0.4, 0.9) | 0.6 (0.4, 0.8) | 0.329 |
|  | Missing | 2 | 10 |  |
| Pretreatment prothrombin time, % | Median (25, 75) | 88.0 (75.1, 103.2) | 98.0 (81.0, 103.3) | 0.492 |
|  | Missing | 13 | 24 |  |
| Pretreatment ICG-R15, % | Median (25, 75) | 11.0 (8.0, no data) | 8.5 (7.0, no data) | 0.400 |
|  | Missing | 34 | 62 |  |
| Preoperative hepatic coma | Yes | 1 (2.7%) | 0 (0.0%) | 0.468 |
|  | Missing | 0 | 21 |  |
| Preoperative ascites | Yes | 3 (8.1%) | 3 (7.0%) | 0.588 |
|  | Missing | 0 | 20 |  |

- Treatable: All tumors could be removed with preservation of a negative surgical margin and sufficient remnant liver volume without performing two-stage hepatectomy with portal vein embolization or major vessel reconstruction. Marginally treatable: All tumors could be removed with preservation of remnant liver volume, regardless of surgical margin, in two-stage hepatectomy with portal vein embolization or major vessel reconstruction. Untreatable: All tumors could not be removed with preservation of sufficient remnant liver volume even if two-stage hepatectomy with portal vein embolization or major vessel reconstruction was performed. Lesions that could be completely ablated were included in the “treatable” category.

CRLM, colorectal liver metastasis; T-Bil, total bilirubin; ICG-R15, indocyanine green retention rate at 15 minutes

**Table S3-1** Clinical findings of the primary lesion of all patients with CRLM

|  | | 2013–2017 | 2005–2007 | *P* value |
| --- | --- | --- | --- | --- |
|  |  | ***n* = 5,085** | ***n* = 3,820** |  |
| Tumor location*† | V | 9 (0.2%) | 5 (0.1%) | 0.155 |
|  | C | 324 (6.5%) | 220 (6.3%) |  |
|  | A | 676 (13.6%) | 488 (14.1%) |  |
|  | T | 441 (8.8%) | 247 (7.1%) |  |
|  | D | 261 (5.2%) | 163 (4.7%) |  |
|  | S | 1,462 (29.4%) | 1,036 (29.9%) |  |
|  | RS | 717 (14.4%) | 492 (14.2%) |  |
|  | Ra | 597 (12.0%) | 465 (13.4%) |  |
|  | Rb | 484 (9.7%) | 343 (9.9%) |  |
|  | P | 13 (0.3%) | 11 (0.3%) |  |
|  | Missing | 101 | 350 |  |
|  | Right side (V, C, A, T) | 1450 (29.1%) | 960 (27.7%) | 0.153 |
|  | Left side (D, S, RS, Ra, Rb, P) | 3,543 (70.9%) | 2,510 (72.3%) |  |
| Macroscopic type* | Type 0 | 52 (1.1%) | 26 (0.8%) | 0.002 |
|  | Type 1 | 215 (4.5%) | 131 (4.3%) |  |
|  | Type 2 | 3,815 (79.8%) | 2,435 (79.4%) |  |
|  | Type 3 | 548 (11.5%) | 412 (13.4%) |  |
|  | Type 4 | 36 (0.8%) | 24 (0.8%) |  |
|  | Type 5 | 112 (2.3%) | 39 (1.3%) |  |
|  | Missing | 307 | 753 |  |
| Tumor size, mm | Median (25, 75%) | 50 (36, 60) | 50 (36, 60) | 0.915 |
|  | Missing | 1142 | 1416 |  |
| Depth of tumor invasion* | M | 4 (0.1%) | 9 (0.3%) | 0.077 |
|  | SM | 65 (1.3%) | 48 (1.4%) |  |
|  | MP | 219 (4.5%) | 130 (3.9%) |  |
|  | SS/A | 2,409 (49.9%) | 1,734 (51.7%) |  |
|  | SE | 1,578 (32.7%) | 1,036 (30.9%) |  |
|  | SI/AI | 555 (11.5%) | 399 (11.9%) |  |
|  | Missing | 255 | 464 |  |
| Lymph node metastasis (N)* | N0 | 1,281 (25.9%) | 1,100 (30.5%) | < 0.001 |
|  | N1 | 1,735 (35.1%) | 1,327 (36.7%) |  |
|  | N2 | 1,204 (24.3%) | 758 (21.0%) |  |
|  | N3 | 487 (9.8%) | 275 (7.6%) |  |
|  | NX | 243 (4.9%) | 152 (4.2%) |  |
|  | Missing | 135 | 208 |  |
| Liver metastasis (H)* | H0 | 1,614 (32.4%) | 1,381 (38.2%) | < 0.001 |
|  | H1 | 1,412 (28.3%) | 908 (25.1%) |  |
|  | H2 | 1,149 (23.1%) | 660 (18.3%) |  |
|  | H3 | 780 (15.7%) | 645 (17.9%) |  |
|  | HX | 26 (0.5%) | 18 (0.5%) |  |
|  | Missing | 104 | 208 |  |
| Peritoneal metastasis (P)* | P0 | 4,306 (87.2%) | 3,218 (89.1%) | < 0.001 |
|  | P1 | 223 (4.5%) | 151 (4.2%) |  |
|  | P2 | 101 (2.0%) | 74 (2.0%) |  |
|  | P3 | 152 (3.1%) | 112 (3.1%) |  |
|  | PX | 155 (3.1%) | 57 (1.6%) |  |
|  | Missing | 148 | 208 |  |
| Distant metastasis excluding liver metastasis and peritoneal metastasis (M) | M0 | 3,409 (68.8%) | 3,035 (84.0%) | < 0.001 |
|  | M1 | 1,516 (30.6%) | 554 (15.3%) |  |
|  | MX | 29 (0.6%) | 23 (0.6%) |  |
|  | Missing | 131 | 208 |  |
| Clinical Stage* | 0 | 8 (0.2%) | 19 (0.5%) | < 0.001 |
|  | I | 114 (2.3%) | 63 (1.8%) |  |
|  | II | 540 (11.0%) | 399 (11.2%) |  |
|  | IIIa | 553 (11.2%) | 492 (13.9%) |  |
|  | IIIb | 315 (6.4%) | 225 (6.3%) |  |
|  | IV | 3,399 (69.0%) | 2,350 (66.2%) |  |
|  | Missing | 156 | 272 |  |

* Described in accordance with General Rules for Clinical and Pathological Studies on Cancer of the Colon, Rectum and Anus (The 7th Edition, Revised Version, January 2009)

† Since it included the cases of multiple cancers, the total percentage may exceed 100%

CRLM, colorectal liver metastasis; I, Ileum; V, Vermiform process; C, Cecum; A, Ascending colon; T, Transverse colon; D, Descending colon; S, Sigmoid colon; RS, Rectosigmoid; Ra, Rectum above the peritoneal reflection; Rb, Rectum below the peritoneal reflection; P, Proctos; Type 0, Superficial type; Type 1, Polypoid type; Type 2, Ulcerated type with clear margin; Type 3, Ulcerated type with infiltration; Type 4, Diffusely infiltrating type; Type 5, Unclassified type; M, Invasion confined to mucosa; SM, Invasion to submucosa; MP, Invasion to muscularis propria; SS/A, Invasion to subserosa or invasion through muscularis propria into pericolic or perirectal tissue; SE, Invasion penetrating serosa; SI/AI, Direct invasion to adjacent organs or structures; N0, No evidence of lymph node metastasis; N1, Metastasis in 1–3 pericolic/perirectal or intermediate lymph nodes; N2, Metastasis in 4 or more pericolic/perirectal or intermediate lymph nodes; N3, Metastasis in the main lymph node(s). In lower rectal cancer, metastasis in the main and/or lateral lymph node(s); NX, Lymph node metastasis cannot be assessed; H0, No liver metastasis; H1, One to 4 metastatic tumors, all of which are 5 cm or less in maximum diameter; H2, Other than H1 or H3; H3, Five or more metastatic tumors at least one of which is more than 5 cm in maximum diameter; HX, Liver metastasis cannot be assessed; P0, No peritoneal metastasis; P1, Metastasis localized to adjacent peritoneum; P2, Limited metastasis to distant peritoneum; P3, Diffuse metastasis to distant peritoneum; PX, Peritoneal metastasis cannot be assessed; M0, No distant metastasis; M1, Distant metastasis; MX, Distant metastasis cannot be assessed

**Table S3-2** Pathological findings of primary lesion of all patients with CRLM (limited to patients who underwent resection)

|  |  | 2013–2017 | 2005–2007 | *P* value |
| --- | --- | --- | --- | --- |
|  |  | ***n* = 4,365** | ***n* = 3,500** |  |
| Depth of tumor invasion* | M | 8 (0.2%) | 4 (0.1%) | 0.149 |
|  | SM | 69 (1.6%) | 53 (1.6%) |  |
|  | MP | 205 (4.8%) | 128 (3.9%) |  |
|  | SS/A | 2,307 (54.5%) | 1,850 (56.0%) |  |
|  | SE | 1,271 (30.0%) | 948 (28.7%) |  |
|  | SI/AI | 374 (8.8%) | 323 (9.8%) |  |
|  | Missing | 131 | 194 |  |
| Histological type* | tub1 | 1,012 (23.6%) | 881 (28.2%) | < 0.001 |
|  | tub2 | 2,922 (68.2%) | 2,001 (64.0%) |  |
|  | por1 | 78 (1.8%) | 104 (3.3%) |  |
|  | por2 | 78 (1.8%) | 28 (0.9%) |  |
|  | muc | 115 (2.7%) | 75 (2.4%) |  |
|  | asc | 0 (0.0%) | 2 (0.1%) |  |
|  | ecc | 11 (0.3%) | 10 (0.3%) |  |
|  | pap | 63 (1.5%) | 24 (0.8%) |  |
|  | scc | 1 (0.0%) | 1 (0.0%) |  |
|  | sig | 5 (0.1%) | 3 (0.1%) |  |
|  | Missing | 80 | 371 |  |
| Lymphatic invasion* | ly0 | 1,304 (30.7%) | 651 (20.0%) | < 0.001 |
|  | ly1 | 1,668 (39.3%) | 1,366 (42.1%) |  |
|  | ly2 | 939 (22.1%) | 923 (28.4%) |  |
|  | ly3 | 335 (7.9%) | 308 (9.5%) |  |
|  | Missing | 119 | 252 |  |
| Venous invasion* | v0 | 638 (15.1%) | 564 (17.5%) | 0.006 |
|  | v1 | 1,386 (32.8%) | 1,087 (33.7%) |  |
|  | v2 | 1,420 (33.6%) | 983 (30.4%) |  |
|  | v3 | 780 (18.5%) | 596 (18.5%) |  |
|  | Missing | 141 | 270 |  |
| Number of lymph node metastases | Median (25, 75%) | 2 (0, 4) | 2 (0, 4) | 0.146 |
|  | Missing | 160 | 521 |  |
| Number of harvested lymph nodes | Median (25, 75%) | 17 (11, 26) | 14 (8, 21) | < 0.001 |
|  | Missing | 277 | 728 |  |
| Tumor deposit | Yes | 390 (15.6%) | 174 (14.3%) | 0.292 |
|  | Missing | 1,860 | 2,279 |  |
| *KRAS* | Mutant | 1055 (41.6%) | 77 (28.5%) | < 0.001 |
|  | Missing | 1,828 | 3,230 |  |

* Described in accordance with General Rules for Clinical and Pathological Studies on Cancer of the Colon, Rectum and Anus (The 7th Edition, Revised Version, January 2009)

CRLM, colorectal liver metastasis; M, Invasion confined to mucosa; SM, Invasion to submucosa; MP, Invasion to muscularis propria; SS/A, Invasion to subserosa or invasion through muscularis propria into pericolic or perirectal tissue; SE, Invasion penetrating serosa; SI/AI, Direct invasion to adjacent organs or structures; tub1, Well differentiated type tubular adenocarcinoma; tub2, Moderately differentiated type tubular adenocarcinoma; por1, Solid type poorly differentiated adenocarcinoma; por2, Non-solid type poorly differentiated adenocarcinoma; muc, Mucinous adenocarcinoma; asc, Adenosquamous carcinoma; ecc, Endocrine cell carcinoma; pap, Papillary adenocarcinoma; scc, Squamous cell carcinoma; sig, Signet-ring cell carcinoma; ly0, No lymphatic invasion; ly1, Minimal lymphatic invasion; ly2, Moderate lymphatic invasion; ly3, Severe lymphatic invasion; v0, No venous invasion; v1, Minimal venous invasion; v2, Moderate venous invasion; v3, Severe venous invasion; EGFR, Endothelial growth factor receptor

**Table S3-3** Clinical findings of the primary lesion of patients who underwent hepatectomy (with/without concomitant ablation therapy) for CRLM

|  | | 2013–2017 | 2005–2007 | *P* value |
| --- | --- | --- | --- | --- |
|  |  | ***n* = 2,788** | ***n* = 2,225** |  |
| Tumor location*† | V | 6 (0.2%) | 2 (0.1%) | 0.057 |
|  | C | 143 (5.2%) | 109 (5.5%) |  |
|  | A | 334 (12.2%) | 251 (12.7%) |  |
|  | T | 249 (9.1%) | 134 (6.8%) |  |
|  | D | 145 (5.3%) | 85 (4.3%) |  |
|  | S | 832 (30.4%) | 611 (31.0%) |  |
|  | RS | 421 (15.4%) | 289 (14.7%) |  |
|  | Ra | 340 (12.4%) | 268 (13.6%) |  |
|  | Rb | 261 (9.5%) | 216 (11.0%) |  |
|  | P | 4 (0.1%) | 6 (0.3%) |  |
|  | Missing | 53 | 254 |  |
|  | Right side (V, C, A, T) | 732 (26.8%) | 496 (25.2%) | 0.218 |
|  | Left side (D, S, RS, Ra, Rb, P) | 2,003 (73.2%) | 1,475 (74.8%) |  |
| Macroscopic type* | Type 0 | 42 (1.6%) | 21 (1.2%) | 0.402 |
|  | Type 1 | 125 (4.7%) | 77 (4.6%) |  |
|  | Type 2 | 2,211 (82.6%) | 1,381 (81.8%) |  |
|  | Type 3 | 248 (9.3%) | 183 (10.8%) |  |
|  | Type 4 | 12 (0.4%) | 8 (0.5%) |  |
|  | Type 5 | 39 (1.5%) | 17 (1.0%) |  |
|  | Missing | 111 | 538 |  |
| Tumor size, mm | Median (25, 75%) | 47 (35, 60) | 45 (32, 60) | 0.059 |
|  | Missing | 584 | 868 |  |
| Depth of tumor invasion* | M | 2 (0.1%) | 9 (0.5%) | 0.077 |
|  | SM | 56 (2.1%) | 41 (2.1%) |  |
|  | MP | 172 (6.3%) | 103 (5.3%) |  |
|  | SS/A | 1,565 (57.5%) | 1,143 (58.9%) |  |
|  | SE | 716 (26.3%) | 472 (24.3%) |  |
|  | SI/AI | 209 (7.7%) | 171 (8.8%) |  |
|  | Missing | 68 | 286 |  |
| Lymph node metastasis (N)* | N0 | 917 (33.5%) | 777 (37.4%) | 0.087 |
|  | N1 | 1,099 (40.2%) | 799 (38.5%) |  |
|  | N2 | 579 (21.2%) | 403 (19.4%) |  |
|  | N3 | 119 (4.4%) | 83 (4.0%) |  |
|  | NX | 21 (0.8%) | 15 (0.7%) |  |
|  | Missing | 53 | 148 |  |
| Liver metastasis (H)* | H0 | 1,176 (42.9%) | 1,079 (51.9%) | < 0.001 |
|  | H1 | 985 (36.0%) | 620 (29.9%) |  |
|  | H2 | 452 (16.5%) | 280 (13.5%) |  |
|  | H3 | 122 (4.5%) | 95 (4.6%) |  |
|  | HX | 4 (0.1%) | 3 (0.1%) |  |
|  | Missing | 49 | 148 |  |
| Peritoneal metastasis (P)* | P0 | 2,644 (97.1%) | 2,008 (96.7%) | 0.793 |
|  | P1 | 53 (1.9%) | 41 (2.0%) |  |
|  | P2 | 16 (0.6%) | 15 (0.7%) |  |
|  | P3 | 6 (0.2%) | 6 (0.3%) |  |
|  | PX | 5 (0.2%) | 7 (0.3%) |  |
|  | Missing | 64 | 148 |  |
| Distant metastasis excluding liver metastasis and peritoneal metastasis (M) | M0 | 2,252 (82.5%) | 1,969 (94.8%) | < 0.001 |
|  | M1 | 470 (17.2%) | 107 (5.2%) |  |
|  | MX | 7 (0.3%) | 1 (0.0%) |  |
|  | Missing | 59 | 148 |  |
| Clinical Stage* | 0 | 5 (0.2%) | 0 (0.0%) | 0.003 |
|  | I | 92 (3.4%) | 58 (2.9%) |  |
|  | II | 439 (16.2%) | 327 (16.4%) |  |
|  | IIIa | 434 (16.0%) | 386 (19.3%) |  |
|  | IIIb | 194 (7.2%) | 168 (8.4%) |  |
|  | IV | 1,547 (57.1%) | 1,057 (53.0%) |  |
|  | Missing | 77 | 229 |  |

* Described in accordance with General Rules for Clinical and Pathological Studies on Cancer of the Colon, Rectum and Anus (The 7th Edition, Revised Version, January 2009)

† Since it included the cases of multiple cancer, the total percentage may exceed 100%

CRLM, colorectal liver metastasis; I, Ileum; V, Vermiform process; C, Cecum; A, Ascending colon; T, Transverse colon; D, Descending colon; S, Sigmoid colon; RS, Rectosigmoid; Ra, Rectum above the peritoneal reflection; Rb, Rectum below the peritoneal reflection; P, Proctos; Type 0, Superficial type; Type 1, Polypoid type; Type 2, Ulcerated type with clear margin; Type 3, Ulcerated type with infiltration; Type 4, Diffusely infiltrating type; Type 5, Unclassified type; M, Invasion confined to mucosa; SM, Invasion to submucosa; MP, Invasion to muscularis propria; SS/A, Invasion to subserosa or invasion through muscularis propria into pericolic or perirectal tissue; SE, Invasion penetrating serosa; SI/AI, Direct invasion to adjacent organs or structures; N0, No evidence of lymph node metastasis; N1, Metastasis in 1–3 pericolic/perirectal or intermediate lymph nodes; N2, Metastasis in 4 or more pericolic/perirectal or intermediate lymph nodes; N3, Metastasis in the main lymph node(s). In lower rectal cancer, metastasis in the main and/or lateral lymph node(s); NX, Lymph node metastasis cannot be assessed; H0, No liver metastasis; H1, One to 4 metastatic tumors, all of which are 5 cm or less in maximum diameter; H2, Other than H1 or H3; H3, Five or more metastatic tumors, at least one of which is more than 5 cm in maximum diameter; HX, Liver metastasis cannot be assessed; P0, No peritoneal metastasis; P1, Metastasis localized to adjacent peritoneum; P2, Limited metastasis to distant peritoneum; P3, Diffuse metastasis to distant peritoneum; PX, Peritoneal metastasis cannot be assessed; M0, No distant metastasis; M1, Distant metastasis; MX, Distant metastasis cannot be assessed

**Table S3-4** Pathological findings of the primary lesions of patients who underwent hepatectomy (with/without concomitant ablation therapy) for CRLM (limited to patients who underwent resection)

|  |  | 2013–2017 | 2005–2007 | *P* value |
| --- | --- | --- | --- | --- |
|  |  | ***n* = 2,757** | ***n* = 2,212** |  |
| Depth of tumor invasion* | M | 5 (0.2%) | 4 (0.2%) | 0.266 |
|  | SM | 61 (2.3%) | 45 (2.2%) |  |
|  | MP | 175 (6.5%) | 105 (5.1%) |  |
|  | SS/A | 1,618 (60.5%) | 1,279 (61.8%) |  |
|  | SE | 637 (23.8%) | 477 (23.1%) |  |
|  | SI/AI | 180 (6.7%) | 159 (7.7%) |  |
|  | Missing | 81 | 143 |  |
| Histological type* | tub1 | 702 (26.0%) | 636 (33.1%) | < 0.001 |
|  | tub2 | 1,854 (68.6%) | 1,191 (62.1%) |  |
|  | por1 | 20 (0.7%) | 35 (1.8%) |  |
|  | por2 | 21 (0.8%) | 8 (0.4%) |  |
|  | muc | 62 (2.3%) | 33 (1.7%) |  |
|  | ecc | 5 (0.2%) | 3 (0.2%) |  |
|  | pap | 38 (1.4%) | 12 (0.6%) |  |
|  | sig | 1 (0.0%) | 1 (0.1%) |  |
|  | Missing | 54 | 293 |  |
| Lymphatic invasion* | ly0 | 924 (34.5%) | 493 (24.3%) | < 0.001 |
|  | ly1 | 1,122 (41.9%) | 902 (44.5%) |  |
|  | ly2 | 517 (19.3%) | 496 (24.5%) |  |
|  | ly3 | 116 (4.3%) | 134 (6.6%) |  |
|  | Missing | 78 | 187 |  |
| Venous invasion* | v0 | 456 (17.1%) | 404 (20.1%) | 0.036 |
|  | v1 | 989 (37.2%) | 731 (36.4%) |  |
|  | v2 | 844 (31.7%) | 584 (29.1%) |  |
|  | v3 | 372 (14.0%) | 291 (14.5%) |  |
|  | Missing | 96 | 202 |  |
| Number of lymph node metastases | Median (25, 75%) | 1 (0, 3) | 1 (0, 3) | 0.969 |
|  | Missing | 105 | 391 |  |
| Number of harvested lymph nodes | Median (25, 75%) | 18 (12, 27) | 15 (9, 23) | < 0.001 |
|  | Missing | 212 | 586 |  |
| Tumor deposit | Yes | 172 (11.5%) | 108 (14.1%) | 0.076 |
|  | Missing | 1,258 | 1,444 |  |
| *KRAS* | Mutant | 563 (38.7%) | 58 (27.5%) | 0.002 |
|  | Missing | 1,301 | 2,001 |  |

* Described in accordance with General Rules for Clinical and Pathological Studies on Cancer of the Colon, Rectum and Anus (The 7th Edition, Revised Version, January 2009)

CRLM, colorectal liver metastasis; M, Invasion confined to mucosa; SM, Invasion to submucosa; MP, Invasion to muscularis propria; SS/A, Invasion to subserosa or invasion through muscularis propria into pericolic or perirectal tissue; SE, Invasion penetrating serosa; SI/AI, Direct invasion to adjacent organs or structures; tub1, Well differentiated type tubular adenocarcinoma; tub2, Moderately differentiated type tubular adenocarcinoma; por1, Solid type poorly differentiated adenocarcinoma; por2, Non-solid type poorly differentiated adenocarcinoma; muc, Mucinous adenocarcinoma; asc, Adenosquamous carcinoma; ecc, Endocrine cell carcinoma; pap, Papillary adenocarcinoma; sig, Signet-ring cell carcinoma; ly0, No lymphatic invasion; ly1, Minimal lymphatic invasion; ly2, Moderate lymphatic invasion; ly3, Severe lymphatic invasion; v0, No venous invasion; v1, Minimal venous invasion; v2, Moderate venous invasion; v3, Severe venous invasion; EGFR, Endothelial growth factor receptor

**Table S3-5** Clinical findings of the primary lesion of patients who underwent ablation therapy alone as local treatment for CRLM

|  | | 2013–2017 | 2005–2007 | *P* value |
| --- | --- | --- | --- | --- |
|  |  | ***n* = 37** | ***n* = 63** |  |
| Tumor location*† | C | 3 (8.1%) | 7 (11.5%) | 0.266 |
|  | A | 6 (16.2%) | 5 (8.2%) |  |
|  | T | 5 (13.5%) | 7 (11.5%) |  |
|  | D | 2 (5.4%) | 6 (9.8%) |  |
|  | S | 7 (18.9%) | 21 (34.4%) |  |
|  | RS | 6 (16.2%) | 4 (6.6%) |  |
|  | Ra | 3 (8.1%) | 8 (13.1%) |  |
|  | Rb | 5 (13.5%) | 3 (4.9%) |  |
|  | Missing | 0 | 2 |  |
|  | Right side (V, C, A, T) | 14 (37.8%) | 19 (31.1%) | 0.218 |
|  | Left side (D, S, RS, Ra, Rb, P) | 23 (62.2%) | 42 (68.9%) |  |
| Macroscopic type* | Type 1 | 1 (2.7%) | 2 (3.5%) | 0.206 |
|  | Type 2 | 35 (94.6%) | 48 (84.2%) |  |
|  | Type 3 | 1 (2.7%) | 7 (12.3%) |  |
|  | Missing | 0 | 6 |  |
| Tumor size, mm | Median (25, 75%) | 50 (34, 62) | 47 (35, 59) | 0.435 |
|  | Missing | 8 | 27 |  |
| Depth of tumor invasion* | SM | 0 (0%) | 1 (1.7%) | 0.432 |
|  | MP | 1 (2.7%) | 3 (5.2%) |  |
|  | SS/A | 22 (59.5%) | 29 (5.0%) |  |
|  | SE | 13 (35.1%) | 19 (32.8%) |  |
|  | SI/AI | 1 (2.7%) | 6 (10.3% |  |
|  | Missing | 0 | 5 |  |
| Lymph node metastasis (N)* | N0 | 917 (33.5%) | 777 (37.4%) | < 0.001 |
|  | N1 | 1099 (40.2%) | 799 (38.5%) |  |
|  | N2 | 579 (21.2%) | 403 (19.4%) |  |
|  | N3 | 119 (4.4%) | 83 (4.0%) |  |
|  | NX | 21 (0.8%) | 15 (0.7%) |  |
|  | Missing | 53 | 148 |  |
| Liver metastasis (H)* | H0 | 21 (56.8%) | 27 (44.3%) | 0.623 |
|  | H1 | 11 (29.7%) | 25 (41.0%) |  |
|  | H2 | 4 (10.8%) | 7 (11.5%) |  |
|  | H3 | 1 (2.7%) | 1 (1.6%) |  |
|  | HX | 0 (0.0%) | 1 (1.6%) |  |
|  | Missing | 0 | 2 |  |
| Peritoneal metastasis (P)* | P0 | 34 (91.9%) | 57 (93.4%) | 0.857 |
|  | P1 | 0 (0.0%) | 1 (1.6%) |  |
|  | P2 | 1 (2.7%) | 1 (1.6%) |  |
|  | P3 | 1 (2.7%) | 1 (1.6%) |  |
|  | PX | 1 (2.7%) | 1 (1.6%) |  |
|  | Missing | 0 | 2 |  |
| Distant metastasis excluding liver metastasis and peritoneal metastasis (M) | M0 | 29 (78.4 | 57 (93.4%) | 0.029 |
|  | M1 | 8 (21.6%) | 3 (4.9%) |  |
|  | MX | 0 (0.0%) | 1 (1.6%) |  |
|  | Missing | 0 | 2 |  |
| Clinical Stage* | I | 0 (0.0%) | 1 (1.8%) | 0.717 |
|  | II | 5 (13.5%) | 10 (16.4%) |  |
|  | IIIa | 7 (18.9%) | 8 (13.1%) |  |
|  | IIIb | 6 (16.2%) | 7 (11.5%) |  |
|  | IV | 19 (51.4%) | 35 (57.4%) |  |
|  | Missing | 0 | 2 |  |

* Described in accordance with General Rules for Clinical and Pathological Studies on Cancer of the Colon, Rectum and Anus (The 7th Edition, Revised Version, January 2009).

† Since it included the cases of multiple cancer, the total percentage may exceed 100%

CRLM, colorectal liver metastasis; C, Cecum; A, Ascending colon; T, Transverse colon; D, Descending colon; S, Sigmoid colon; RS, Rectosigmoid; Ra, Rectum above the peritoneal reflection; Rb, Rectum below the peritoneal reflection; Type 1, Polypoid type; Type 2, Ulcerated type with clear margin; Type 3, Ulcerated type with infiltration; SM, Invasion to submucosa; MP, Invasion to muscularis propria; SS/A, Invasion to subserosa or invasion through muscularis propria into pericolic or perirectal tissue; SE, Invasion penetrating serosa; SI/AI, Direct invasion to adjacent organs or structures; N0, No evidence of lymph node metastasis; N1, Metastasis in 1–3 pericolic/perirectal or intermediate lymph nodes; N2, Metastasis in 4 or more pericolic/perirectal or intermediate lymph nodes; N3, Metastasis in the main lymph node(s). In lower rectal cancer, metastasis in the main and/or lateral lymph node(s); H0, No liver metastasis; H1, One to 4 metastatic tumors, all of which are 5 cm or less in maximum diameter; H2, Other than H1 or H3; H3, Five or more metastatic tumors, at least one of which is more than 5 cm in maximum diameter; P0, No peritoneal metastasis; P1, Metastasis localized to adjacent peritoneum; P3, Diffuse metastasis to distant peritoneum; PX, Peritoneal metastasis cannot be assessed; M0, No distant metastasis; M1, Distant metastasis

**Table S3-6** Pathological findings of the primary lesion of patients who underwent ablation therapy alone as local treatment for CRLM (limited to patients who underwent resection)

|  |  | 2013–2017 | 2005–2007 | *P* value |
| --- | --- | --- | --- | --- |
|  |  | ***n* = 36** | ***n* = 61** |  |
| Depth of tumor invasion* | MP | 1 (2.9%) | 4 (6.9%) | 0.851 |
|  | SS/A | 21 (60.0%) | 34 (58.6%) |  |
|  | SE | 10 (28.6%) | 15 (25.9%) |  |
|  | SI/AI | 3 (8.6%) | 5 (8.6%) |  |
|  | Missing | 1 | 3 |  |
| Histological type* | tub1 | 8 (22.2%) | 21 (36.2%) | 0.353 |
|  | tub2 | 27 (75.0%) | 35 (60.3%) |  |
|  | por2 | 1 (2.8%) | 1 (1.7%) |  |
|  | pap | 0 (0.0%) | 1 (1.7%) |  |
|  | Missing | 0 | 3 |  |
| Lymphatic invasion* | ly0 | 8 (22.2%) | 8 (13.8%) | 0.630 |
|  | ly1 | 16 (44.4%) | 24 (41.4%) |  |
|  | ly2 | 10 (27.8%) | 21 (36.2%) |  |
|  | ly3 | 2 (5.6%) | 5 (8.6%) |  |
|  | Missing | 0 | 3 |  |
| Venous invasion* | v0 | 2 (5.9%) | 10 (17.2%) | 0.040 |
|  | v1 | 9 (26.5%) | 26 (44.8%) |  |
|  | v2 | 18 (52.9%) | 16 (27.6%) |  |
|  | v3 | 5 (14.7%) | 6 (10.3%) |  |
|  | Missing | 2 | 3 |  |
| Number of lymph node metastases | Median (25, 75%) | 1 (0, 4) | 1 (0, 3) | 0.783 |
|  | Missing | 1 | 8 |  |
| Number of harvested lymph nodes | Median (25, 75%) | 15 (9, 25) | 14 (9, 20) | 0.196 |
|  | Missing | 1 | 8 |  |
| Tumor deposit | Yes | 4 (16.7%) | 11 (7.1%) | 0.381 |
|  | Missing | 12 | 47 |  |
| *KRAS* | Mutant | 9 (56.3%) | 2 (50.0%) | 0.625 |
|  | Missing | 20 | 57 |  |

* Described in accordance with General Rules for Clinical and Pathological Studies on Cancer of the Colon, Rectum and Anus (The 7th Edition, Revised Version, January 2009)

CRLM, colorectal liver metastasis; SM, Invasion to submucosa; MP, Invasion to muscularis propria; SS/A, Invasion to subserosa or invasion through muscularis propria into pericolic or perirectal tissue; SE, Invasion penetrating serosa; SI/AI, Direct invasion to adjacent organs or structures; tub1, Well differentiated type tubular adenocarcinoma; tub2, Moderately differentiated type tubular adenocarcinoma; por2, Non-solid type poorly differentiated adenocarcinoma; pap, muc, Mucinous adenocarcinoma; pap, Papillary adenocarcinoma; ly0, No lymphatic invasion; ly1, Minimal lymphatic invasion; ly2, Moderate lymphatic invasion; ly3, Severe lymphatic invasion; v0, No venous invasion; v1, Minimal venous invasion; v2, Moderate venous invasion; v3, Severe venous invasion; EGFR, Endothelial growth factor receptor

**Table S4** Outcomes after ablation therapy alone as local treatment for CRLM

|  |  | 2013–2017 | 2005–2007 | *P* value |
| --- | --- | --- | --- | --- |
|  |  | ***n* = 37** | ***n* = 63** |  |
| Final number of CRLMs | Median (25, 75%) | 1 (1, 2) | 1 (1, 2) | 0.051 |
|  | Missing | 5 | 5 |  |
| Maximum diameter of CRLM, mm | Median (25, 75%) | 18 (13, 20) | 20 (14, 33) | 0.189 |
|  | Missing | 6 | 9 |  |
| Postoperative complication (Clavien-Dindo classification ≥ III) | Yes | 1 (3.1%) | 2 (3.5%) | 0.707 |
|  | Missing | 5 | 8 |  |

¶ NAFLD activity was graded using Kleiner’s NAFLD activity score as follows: 0, absent; 1–4, mild; 5–, severe

¶¶ Steatosis was estimated as the percentage of involved hepatocytes, and was categorized as follows: 0, absent; 1, mild (steatosis in <30% of the hepatocytes); 2, moderate (steatosis in 30–60% of the hepatocytes); 3, severe (steatosis in >60% of the hepatocytes)

¶¶¶ Sinusoidal dilatation was graded semi-quantitatively using Rubbia Brandt’s grading as follows: 0, absent; 1, mild (centrilobular involvement limited to one-third of the lobular surface); 2, moderate (centrilobular involvement extending in two-thirds of the lobular surface); 3, severe (complete lobular involvement).

CRLM, colorectal liver metastasis; NAFLD, non-alcoholic steatohepatitis

**Table S5-1** Time series data of tumor marker values (all patients with CRLM)

|  | | 2013–2017 | 2005–2007 | *P* value |
| --- | --- | --- | --- | --- |
|  |  | ***n* = 5,085** | ***n* = 3,820** |  |
| CA19-9, U/mL, Median (25, 75%) | At diagnosis of primary lesion | 24.2 (8.0, 150.2) | 27.0 (8.1, 164.0) | 0.476 |
|  | Missing | 585 | 1,197 |  |
|  | After resection of primary lesion | 13.0 (5.4, 46.2) | 15.0 (6.0, 61.5) | 0.001 |
|  | Missing | 1,385 | 1,787 |  |
|  | Before treatment for CRLM* | 28.0 (8.9, 187.0) | 28.4 (8.8, 158.1) | 0.213 |
|  | Missing | 1,237 | 1,670 |  |
|  | Lowest value throughout chemotherapy | 29.2 (9.0, 229.4) | 31.0 (9.0, 206.0) | 0.951 |
|  | Missing | 3,601 | 3,111 |  |
| CEA, ng/mL, Median (25, 75%) | At diagnosis of primary lesion | 15.8 (4.5, 91.4) | 16.7 (4.4, 90.6) | 0.667 |
|  | Missing | 521 | 1,082 |  |
|  | After resection of primary lesion | 4.1 (2.0, 19.8) | 4.5 (1.9, 31.3) | 0.075 |
|  | Missing | 1,343 | 1,667 |  |
|  | Before treatment for CRLM* | 17.0 (4.9, 109.8) | 17.0 (5.0, 84.3) | 0.261 |
|  | Missing | 1,200 | 1,607 |  |
|  | Lowest value throughout chemotherapy | 12.9 (4.1, 81.6) | 19.2 (4.1, 117.9) | 0.032 |
|  | Missing | 3,561 | 3,070 |  |

* The value before treatment for CRLM was defined as the same as the value at diagnosis of the primary lesion in patients who underwent simultaneous resection of the primary lesion and CRLM

CRLM, colorectal liver metastasis; CA19-9, carbohydrate antigen 19-9; CEA, carcinoembryonic antigen

**Table S5-2** Time series data of tumor marker values (patients who underwent hepatectomy (with/without concomitant ablation therapy) for CRLM)

|  | | 2013–2017 | 2005–2007 | *P* value |
| --- | --- | --- | --- | --- |
|  |  | ***n* = 2,788** | ***n* = 2,225** |  |
| CA19-9, U/mL, Median (25, 75%) | At diagnosis of primary lesion | 15.1 (6.4, 44.6) | 17.0 (6.0, 54.0) | 0.182 |
|  | Missing | 405 | 1074 |  |
|  | After resection of primary lesion | 10.7 (5.0, 24.0) | 11.0 (5.0, 26.1) | 0.747 |
|  | Missing | 483 | 885 |  |
|  | Before treatment for CRLM* | 17.6 (7.1, 56.7) | 20.0 (7.9, 70.0) | 0.047 |
|  | Missing | 536 | 890 |  |
|  | Lowest value throughout chemotherapy | 13.8 (6.3, 37.0) | 10.1 (2.7, 19.5) | 0.107 |
|  | Missing | 2712 | 2212 |  |
|  | Before hepatectomy** | 16.0 (7.1, 39.3) | 22.6 (8.0, 74.1) | < 0.001 |
|  | Missing | 224 | 468 |  |
|  | After hepatectomy | 10.7 (5.0, 20.3) | 12.2 (5.9, 25.0) | < 0.001 |
|  | Missing | 402 | 749 |  |
| CEA, ng/mL, Median (25, 75%) | At diagnosis of primary lesion | 8.7 (3.3, 33.2) | 8.9 (3.3, 34.7) | 0.905 |
|  | Missing | 368 | 807 |  |
|  | After resection of primary lesion | 3.0 (1.7, 7.1) | 2.7 (1.6, 6.5) | 0.027 |
|  | Missing | 449 | 987 |  |
|  | Before treatment for CRLM* | 9.6 (3.7, 36.0) | 11.9 (4.1, 47.15) | 0.016 |
|  | Missing | 521 | 843 |  |
|  | Lowest value throughout chemotherapy | 4.0 (2.4, 6.6) | 1.9 (1.6, 4.1) | 0.024 |
|  | Missing | 2708 | 2212 |  |
|  | Before hepatectomy** | 7.0 (3.2, 22.6) | 11.4 (4.0, 44.1) | < 0.001 |
|  | Missing | 206 | 398 |  |
|  | After hepatectomy | 2.5 (1.5, 4.2) | 2.7 (1.6, 5.1) | 0.006 |
|  | Missing | 377 | 667 |  |

* The value before treatment for CRLM was defined as the same as the value at diagnosis of the primary lesion in patients who underwent simultaneous resection of the primary lesion and CRLM.

** The value before hepatectomy was defined as the same as the value before treatment for CRLM in patients without preoperative chemotherapy.

CRLM, colorectal liver metastasis; CA19-9, carbohydrate antigen 19-9; CEA, carcinoembryonic antigen

**Table S5-3** Time series data of tumor marker values (patients who underwent ablation therapy alone as local treatment for CRLM)

|  | | 2013–2017 | 2005–2007 | *P* value |
| --- | --- | --- | --- | --- |
|  |  | ***n* = 37** | ***n* = 63** |  |
| CA19-9, U/mL, Median (25, 75%) | At diagnosis of primary lesion | 20.7 (7.0, 63.4) | 27.8 (7.6, 109.3) | 0.701 |
|  | Missing | 3 | 11 |  |
|  | After resection of primary lesion | 17.3 (5.8, 24.9) | 24.5 (6.7, 67.5) | 0.238 |
|  | Missing | 7 | 23 |  |
|  | Before treatment for CRLM* | 29.0 (13.8, 13.6) | 15.2 (6.1, 50.4) | 0.233 |
|  | Missing | 4 | 25 |  |
|  | Lowest value throughout chemotherapy | 17.6 (7.2, 1599.51) | 4.0 (4.0, 4.0) | 0.571 |
|  | Missing | 31 | 62 |  |
|  | Pretreatment** | 27.7 (12.1, 99.4) | 15.2 (6.1, 50.4) | 0.307 |
|  | Missing | 3 | 25 |  |
|  | Post-treatment | 14.5 (7.0, 28.8) | 28.2 (7.6, 52.4) | 0.174 |
|  | Missing | 11 | 23 |  |
| CEA, ng/mL, Median (25, 75%) | At diagnosis of primary lesion | 9.6 (5.5, 32.0) | 9.7　(4.7, 29.9) | 0.498 |
|  | Missing | 1 | 10 |  |
|  | After resection of primary lesion | 4.3 (2.3, 10.2) | 3.6 (2.0, 15.7) | 0.749 |
|  | Missing | 7 | 22 |  |
|  | Before treatment for CRLM* | 11.4 (4.6, 49.7) | 7.4 (3.4, 16.3) | 0.117 |
|  | Missing | 5 | 25 |  |
|  | Lowest value throughout chemotherapy | 4.1 (2.2, 10.8) | 3.3 (3.3, 3.3) | 1.000 |
|  | Missing | 31 | 62 |  |
|  | Pretreatment** | 6.1 (3.8, 23.9) | 7.5 (3.3, 23.5) | 0.892 |
|  | Missing | 4 | 20 |  |
|  | Post-treatment | 4.7 (3.0, 7.8) | 5.2 (2.3, 22.6) | 0.762 |
|  | Missing | 11 | 22 |  |

* The value before treatment for CRLM was defined as the same as the value at diagnosis of the primary lesion in patients who underwent simultaneous resection of the primary lesion and CRLM

** The value before hepatectomy was defined as the same as the value before treatment for CRLM in patients without preoperative chemotherapy

CRLM, colorectal liver metastasis; CA19-9, carbohydrate antigen 19-9; CEA, carcinoembryonic antigen

**Table S6** Implementation status of chemotherapy

|  |  | 2013–2017 | 2005–2007 | *P* value |
| --- | --- | --- | --- | --- |
| Neoadjuvant chemotherapy for CRLM (regardless of implementation of local treatment) |  | 623 (12.3%) | 130 (3.4%) | < 0.001 |
| Regimen | Oral FU regimens | 3 (0.5%) | 13 (10.5%) | < 0.001 |
|  | L-OHP regimens | 515 (83.7%) | 62 (50.0%) |  |
|  | CPT-11 regimens | 55 (8.9%) | 16 (12.9%) |  |
|  | Triplet regimens | 14 (2.4%) | 0 (0.0%) |  |
|  | 5-FU/LV | 3 (0.5%) | 13 (10.5%) |  |
|  | HAI | 0 (0.0%) | 10 (8.1%) |  |
|  | Missing | 8 | 6 |  |
| Molecular targeted agent | Bevacizumab | 341 (54.7%) | 7 (5.4%) | < 0.001 |
|  | Cetuximab | 46 (7.4%) | 0 (0.0%) |  |
|  | Panitumumab | 78 (12.5%) | 0 (0.0%) |  |
|  | None or Missing | 158 (25.4%) | 123 (94.6%) |  |
| Patients with resected primary lesion out of the patients with metachronous liver metastasis |  | 1,687 (98.0%) | 1,390 (98.6%) | 0.233 |
| Adjuvant chemotherapy after resection of the primary lesion | Performed | 848 (51.5%) | 728 (57.2%) | 0.002 |
|  | Missing | 39/1687 | 117/1390 |  |
| Regimen | Oral FU regimens | 523 (62.6%) | 406 (60.4%) | < 0.001 |
|  | L-OHP regimens | 289 (34.6%) | 43 (6.4%) |  |
|  | CPT-11 regimens | 10 (1.2%) | 10 (1.5%) |  |
|  | 5-FU/LV | 13 (1.6%) | 146 (21.7%) |  |
|  | Others | 0 (0.0%) | 2 (0.3%) |  |
|  | Missing | 13/848 | 55/728 |  |
| Molecular targeted agent | Bevacizumab | 33 (2.0%) | 0 (0.0%) | < 0.001 |
|  | Cetuximab | 5 (0.3%) | 0 (0.0%) |  |
|  | Panitumumab | 11 (0.7%) | 0 (0.0%) |  |
|  | None or Missing | 1,638 (97.1%) | 1,390 (100.0%) |  |
| Patients who underwent local treatment for CRLM* |  | 2,821 (55.5%) | 2,287 (59.9%) | < 0.001 |
| Chemotherapy for CRLM before local treatment (regardless of intention to perform local treatment after the chemotherapy) | Performed | 1,044 (37.0%) | 499 (21.8%) | < 0.001 |
| Chemotherapy as a neoadjuvant |  | 587 (20.8%) | 116 (5.1%) | < 0.001 |
| Regimen | Oral FU regimens | 59 (5.7%) | 108 (22.1%) | < 0.001 |
|  | L-OHP regimens | 839 (81.1%) | 211 (43.1%) |  |
|  | CPT-11 regimens | 105 (10.2%) | 50 (10.2%) |  |
|  | Triplet regimens | 23 (2.2%) | 0 (0.0%) |  |
|  | 5-FU/LV | 7 (0.7%) | 49 (10.0%) |  |
|  | HAI | 2 (0.2%) | 70 (14.3%) |  |
|  | Others | 0 (0.0%) | 1 (0.2%) |  |
|  | Missing | 9 | 10 |  |
| Molecular targeted agent | Bevacizumab | 535 (19.0%) | 21 (0.9%) | < 0.001 |
|  | Cetuximab | 93 (3.3%) | 0 (0.0%) |  |
|  | Panitumumab | 154 (5.5%) | 0 (0.0%) |  |
|  | None or Missing | 2,039 (72.3%) | 2,266 (99.1%) |  |
| Patients who underwent hepatectomy |  | 2,788 (54.8%) | 2,225 (58.2%) | 0.001 |
| Adjuvant chemotherapy after hepatectomy | Performed | 1,382 (50.7%) | 1,443 (67.2%) | < 0.001 |
|  | Missing | 63 | 77 |  |
| Regimen | Oral FU regimens | 435 (32.2%) | 651 (46.2%) | < 0.001 |
|  | L-OHP regimens | 769 (56.9%) | 446 (31.6%) |  |
|  | CPT-11 regimens | 110 (8.1%) | 91 (6.5%) |  |
|  | Triplet regimens | 2 (0.1%) | 0 (0.0%) |  |
|  | 5-FU/LV | 35 (2.6%) | 140 (9.9%) |  |
|  | HAI | 0 (0.0%) | 79 (5.6%) |  |
|  | Missing | 31 | 33 |  |
| Molecular targeted agent | Bevacizumab | 176 (6.3%) | 24 (1.1%) | < 0.001 |
|  | Cetuximab | 19 (0.7%) | 3 (0.1%) |  |
|  | Panitumumab | 37 (1.3%) | 1 (0.0%) |  |
|  | None or Missing | 2,556 (91.7%) | 2,197 (98.7%) |  |
| Patients who did not undergo local treatment for CRLM |  | 2,264 (44.5%) | 1,533 (40.1%) | < 0.001 |
| Patients who underwent chemotherapy | Performed | 1276 (56.4%) | 815 (53.2%) | 0.052 |
|  | Missing | 679 | 520 |  |
| Regimen | Oral FU regimens | 124 (9.8%) | 149 (18.3%) | < 0.001 |
|  | L-OHP regimens | 966 (76.4%) | 376 (46.2%) |  |
|  | CPT-11 regimens | 130 (10.3%) | 142 (17.4%) |  |
|  | Triplet regimens | 14 (1.1%) | 0 (0.0%) |  |
|  | 5-FU/LV | 26 (2.1%) | 53 (6.5%) |  |
|  | HAI | 3 (0.2%) | 93 (11.4%) |  |
|  | Others | 2 (0.2%) | 1 (0.1%) |  |
|  | Missing | 11 | 1 |  |
| Molecular targeted agent | Bevacizumab | 675 (29.8%) | 45 (2.9%) | < 0.001 |
|  | Cetuximab | 73 (3.2%) | 0 (0.0%) |  |
|  | Panitumumab | 171 (7.6%) | 0 (0.0%) |  |
|  | Regorafenib | 1 (0.0%) | 0 (0.0%) |  |
|  | Nivolumab | 1 (0.0%) | 0 (0.0%) |  |
|  | None or Missing | 1,343 (59.3%) | 1,488 (97.1%) |  |
| Chemotherapy for CRLM excluding adjuvant chemotherapy after hepatectomy |  | 2,320 (1,044+1,276) (45.6%) | 1,314 (499+815) (34.4%) | < 0.001 |
| Overall response of chemotherapy (RECIST criteria**) | Missing | 488/2,320 | 605/1,314 |  |
| in 1,583 (=1,999–416) | CR | 76/1,832 (4.1%) | 30/708 (4.2%) | < 0.001 |
|  | PR | 809/1,832 (44.2%) | 235/708 (33.2%) |  |
|  | SD | 683/1,832 (37.3%) | 245/708 (34.6%) |  |
|  | PD | 233/1,832 (12.7%) | 173/708 (24.4%) |  |
|  | NE | 31/1,832 (1.7%) | 25/708 (3.5%) |  |

Oral FU regimens included UFT (tegafur uracil) & LV (leucovorin), Capecitabine, S-1 (tegafur gimeracil oteracil), Trifluridine/Tipiracil, and UFT alone; L-OHP regimens included FOLFOX (5-fluorouracil/levofolinate/oxaliplatin), CapeOX (capecitabine/oxaliplatin), and SOX (S-1/oxaliplatin); CPT-11 regimens include FOLFIRI (5-fluorouracil/levofolinate/irinotecan), and IRIS (irinotecan/S-1); Triplet regimen included FOLFOXIRI (5-fluorouracil/levofolinate/oxaliplatin/irinotecan).

CRLM, colorectal liver metastasis; 5-FU, 5-fluorouracil; HAI, Hepatic artery infusion chemotherapy

* Local treatment for CRLM included hepatectomy and ablation therapy (radiofrequency ablation and microwave coagulation therapy), but not hepatic arterial infusion chemotherapy

** RECIST, Response Evaluation Criteria in Solid Tumors; CR, Complete Response; PR, Partial Response; SD, Stable Disease; PD, Progressive Disease; NE, Not Evaluable

**Table S7** Recurrence after hepatectomy (with/without concomitant ablation therapy) for CRLM

|  |  | 2013–2017 | 2005–2007 | *P* value |
| --- | --- | --- | --- | --- |
|  |  | ***n* = 2,788** | ***n* = 2,225** |  |
| Total recurrence |  | 1,647 (59.1%) | 1,436 (64.5%) | < 0.001 |
|  | Missing | 146 | 122 |  |
| Intrahepatic recurrence of remnant liver |  | 1,049 (66.2%) | 935 (66.0%) | 0.930 |
|  | Not cut-end recurrence | 829 (85.6%) | 696 (87.7%) | 0.217 |
|  | Cut-end recurrence | 167 (17.2%) | 134 (17.0%) | 0.924 |
| Extrahepatic recurrence |  | 1,188 (72.6%) | 1,056 (73.5%) | 0.547 |
| Treatment for recurrence of remnant liver | Re-hepatectomy for intrahepatic recurrence | 416 (41.2%) | 333 (38.0%) | 0.068 |
|  | Ablation for intrahepatic recurrence | 31 (3.1%) | 40 (4.6%) |  |
|  | Radiation | 10 (1.0%) | 6 (0.7%) |  |
|  | Chemotherapy or  Best supportive care | 550 (54.5%) | 498 (56.8%) |  |
|  | Missing | 39 | 58 |  |

CRLM, colorectal liver metastasis

**Table S8** The list of the departments or institutions that registered data of patients with CRLM

| Hokkaido, Tohoku | Hokkaido | National Hospital Organization Hokkaido Cancer Center | Department of Gastroenterological Surgery |
| --- | --- | --- | --- |
|  | Hokkaido | Sapporo Medical University | Department of Surgery, Surgical Oncology and Science |
|  | Hokkaido | Sapporo Medical University School of Medicine | Department of Gastroenterology and Hepatology |
|  | Hokkaido | Kin-ikyo Chuo Hospital | Department of Surgery |
|  | Hokkaido | Hokkaido University Faculty of Medicine | Department of Gastroenterological Surgery II |
|  | Hokkaido | Hokkaido University | Department of Gastroenterological Surgery I |
|  | Hokkaido | Sapporo Kosei General Hospital | Department of Surgery |
|  | Aomori | Hirosaki University Graduate School of Medicine | Department of Gastroenterological Surgery |
|  | Akita | Akita University Graduate School of Medicine | Department of Gastroenterological Surgery |
|  | Akita | Akita Municipal Hospital | Department of Surgery |
|  | Iwate | Iwate Medical University | Department of Surgery |
|  | Iwate | Iwate Prefectural Central Hospital | Department of Gastroenterological Surgery |
|  | Miyagi | Miyagi Cancer Center | Department of Digestive Surgery |
|  | Miyagi | Tohoku University Hospital | Surgery |
|  | Fukushima | Fukushima Medical University | Department of Gastrointestinal Tract Surgery |
|  | Yamagata | Yamagata University Hospital | First Department of Surgery |
| Kanto | Gunma | Gunma University Graduate School of Medicine | Department of General Surgical Science |
|  | Tochigi | Jichi Medical University Hospital | Department of Surgery |
|  | Tochigi | Tochigi Cancer Center | Colorectal Surgery |
|  | Tochigi | Dokkyo Medical University | First Department of Surgery |
|  | Tochigi | Saiseikai Utsunomiya Hospital | Department of Surgery |
|  | Ibaraki | Ibaraki Prefectural Central Hospital | Department of Surgery |
|  | Saitama | Saitama Cancer Center | Department of Gastroenterological Surgery |
|  | Saitama | National Defense Medical College | Department of Surgery |
|  | Saitama | Fukaya Red Cross Hospital | Department of Surgery |
|  | Saitama | Saitama Medical University Saitama Medical Center | Department of Digestive Tract and General Surgery |
|  | Saitama | Saitama Citizens Medical Center | Department of Surgery |
|  | Chiba | Kimitsu Chuo Hospital | Department of Surgery |
|  | Chiba | Chiba Cancer Center | Department of Gastroenterological Surgery |
|  | Chiba | Teikyo University Chiba Medical Center | Department of Surgery |
|  | Chiba | Tokyo Dental College Ichikawa General Hospital | Department of Surgery |
|  | Tokyo | Tokyo Metropolitan Cancer and Infectious Diseases Center Komagome Hospital | Department of Surgery |
|  | Tokyo | Graduate School of Medicine, University of Tokyo | Hepato-Biliary-Pancreatic Surgery Division, Department of Surgery |
|  | Tokyo | Tokyo Medical and Dental University | Gastrointestinal Surgery |
|  | Tokyo | Cancer Institute Hospital, Japanese Foundation for Cancer Research | Department of Hepatobiliary Pancreatic Surgery |
|  | Tokyo | Nippon Medical School | Department of Gastrointestinal and Hepato-Biliary-Pancreatic Surgery |
|  | Tokyo | Toho University Faculty of Medicine | Department of Surgery, Division of Gastroenterological Surgery (Omori) |
|  | Tokyo | Teikyo University School of Medicine | Department of Surgery |
|  | Tokyo | Japan Community Healthcare Organization Tokyo Shinjuku Medical Center | Department of Surgery |
|  | Tokyo | The University of Tokyo | Department of Surgical Oncology |
|  | Tokyo | Tokyo Women’s Medical University | Institute of Gastroenterology, Department of Surgery |
|  | Tokyo | Juntendo University Faculty of Medicine | Department of Coloproctological Surgery |
|  | Tokyo | Omori Red Cross Hospital | Department of Surgery |
|  | Tokyo | Showa University Hospital | Department of Surgery, Division of General and Gastroenterological Surgery |
|  | Tokyo | Toho University Ohashi Medical Center | Department of Surgery |
|  | Tokyo | Kyorin University, School of Medicine | Department of Colorectal Surgery |
|  | Tokyo | Mitsui Memorial Hospital | Department of Gastroenterological Surgery |
|  | Tokyo | Tokyo Kyosai Hospital | Department of Surgery |
|  | Tokyo | JSDF Central Hospital | Department of Surgery |
|  | Tokyo | Tokyo Medical and Dental University | Department of Hepatobiliary & Pancreatic Surgery |
|  | Tokyo | Keio University | Department of Surgery |
|  | Tokyo | International University of Health and Welfare Mita Hospital | Digestive Diseases Center |
|  | Tokyo | Tokyo Yamate Medical Center | Department of Coloproctology |
|  | Tokyo | Tokyo Metropolitan Hiroo Hospital | Surgery |
|  | Tokyo | Tokyo Women’s Medical University Yachiyo Medical Center | Division of Gastroenterological Surgery |
|  | Tokyo | The Jikei University School of Medicine | Department of Surgery |
|  | Kanagawa | Kawasaki Municipal Ida Hospital | Department of Surgery |
|  | Kanagawa | Kitasato University | Department of Colorectal Surgery, School of Medicine |
|  | Kanagawa | St. Marianna University School of Medicine | Division of gastroenterology and general surgery |
|  | Kanagawa | Yokohama City University Graduate School of Medicine | Department of Gastroenterological Surgery |
|  | Kanagawa | Kanagawa Cancer Center | Department of Gastrointestinal Surgery |
|  | Kanagawa | Showa University Fujigaoka Hospital | General and Gastroenterological Surgery |
|  | Kanagawa | Yokohama Asahi Chuo General Hospital | Department of Surgery |
| Hokuriku, Koushinetsu | Niigata | Nagaoka Chuo General Hospital | Department of Surgery |
|  | Niigata | Niigata University Graduate School of Medical and Dental Sciences | Division of Digestive and General Surgery |
|  | Niigata | Niigata City General Hospital | Digestive Surgery |
|  | Ishikawa | Ishikawa Prefectural Central Hospital | Gastroenterological Surgery |
|  | Ishikawa | Kanazawa University Graduate School of Medicine | Department of Gastroenterological Surgery |
|  | Ishikawa | Kanazawa Medical University | Department of General and Digestive Surgery |
|  | Toyama | Toyama Prefectural Central Hospital |  |
|  | Fukui | Fukui Prefecture-Saiseikai Hospital | Department of Surgery |
|  | Fukui | University of Fukui | First Department of Surgery |
|  | Yamanashi | University of Yamanashi, Faculty of Medicine | First Department of Surgery |
| Tokai | Shizuoka | Seirei Hamamatsu General Hospital | Department of Coloproctology |
|  | Shizuoka | Matsuda Hospital | Colo-proctological Institute |
|  | Shizuoka | Shizuoka General Hospital | Department of Surgery |
|  | Shizuoka | Hamamatsu University School of Medicine | Second Department of Surgery |
|  | Shizuoka | Iwata City Hospital | Department of Gastroenterological Surgery |
|  | Aichi | Fujita Health University | Surgery |
|  | Aichi | Kariya Toyota General Hospital | Department of Gastrointestinal Surgery |
|  | Aichi | Japanese Red Cross Nagoya Daiichi Hospital | Department of Surgery |
|  | Aichi | School of Medicine, Fujita Health University Banbuntane Hotokukai Hospital | Department of Gastroenterological Surgery |
|  | Aichi | Aichi Medical University | Department of Gastroenterological Surgery |
|  | Aichi | Toyohashi Municipal Hospital | Department of general surgery |
|  | Gifu | Gifu Prefectural Tajimi Hospital | Department of Surgery |
|  | Gifu | Matsunami General Hospital | Department of Surgery |
|  | Gifu | Gifu University Graduate School of Medicine / School of Medicine | Department of Surgical Oncology |
|  | Gifu | Gifu Prefectural General Medical Center | Department of Surgery |
|  | Gifu | Ogaki Municipal Hospital | Department of Surgery |
|  | Mie | Mie University | Department of Gastrointestinal and Pediatric Surgery |
|  | Mie | Mie University Graduate School of Medicine | Hepatobiliary Pancreatic and Transplant Surgery |
| Kansai | Shiga | Shiga General Hospital | Department of Surgery |
|  | Shiga | Japanese Red Cross Otsu Hospital | Department of Surgery |
|  | Kyoto | Kyoto Prefectural University of Medicine | Department of Surgery, Division of Digestive Surgery |
|  | Kyoto | Japanese Red Cross Society Kyoto Daiichi Hospital | Department of Hepato-Pancreatic Surgery |
|  | Kyoto | Japanese Red Cross Kyoto Daini Hospital | Department of Surgery |
|  | Kyoto | National Hospital Organization Kyoto Medical Center | Department of Surgery |
|  | Osaka | Kindai University Faculty of Medicine | Department of Surgery |
|  | Osaka | Kindai University Faculty of Medicine | Critical Care Medical Center |
|  | Osaka | National Hospital Organization Osaka National Hospital | Department of Hepato-Biliary-Pancreatic Surgery |
|  | Osaka | Bell Land General Hospital | Department of Surgery |
|  | Osaka | Graduate School of Medicine, Osaka University | Department of Gastroenterological Surgery |
|  | Osaka | Toyonaka Municipal Hospital | Department of Surgery |
|  | Osaka | Osaka Rosai Hospital | Dpt. of Surgery |
|  | Osaka | Osaka City General Hospital | Hepato-Biliary-Pancreatic Surgery |
|  | Osaka | Osaka Metropolitan University Graduate School of Medicine | Department of Hepato-Biliary-Pancreatic Surgery |
|  | Osaka | Osaka Metropolitan University Graduate School of Medicine | Department of Surgical Oncology |
|  | Osaka | Osaka Medical College | Department of General and Gastroenterological Surgery |
|  | Osaka | Suita Municipal Hospital | Dept. of Surgery |
|  | Osaka | Rinku General Medical Center | Department of Surgery |
|  | Osaka | Kansai Medical University | Department of surgery |
|  | Osaka | Sakai City Medical Center | Department of Surgery |
|  | Osaka | Saiseikai Suita Hospital | Department of Surgery |
|  | Hyogo | Meiwa Hospital | Department of Surgery |
|  | Hyogo | Kinki Central Hospital | Surgery |
|  | Hyogo | Hyogo College of Medicine | Division of Lower GI Surgery, Department of Surgery |
|  | Hyogo | Himeji Central Hospital | Department of Gastroenterol Surgery |
|  | Hyogo | Kobe University Graduate School of Medicine | Department of Surgery, Division of Hepato-Biliary-Pancreatic Surgery |
|  | Hyogo | Kansai Rosai Hospital | Department of Surgery |
|  | Hyogo | Hyogo Cancer Center | Gastroenterological Surgery |
|  | Hyogo | Shinko Memorial Hospital | Department of Surgery |
|  | Nara | Nara Medical University | Department of Surgery |
|  | Nara | Dongo Hospital | Department of Surgery |
|  | Wakayama | Wakayama Medical University | Second Department of Surgery |
| Chugoku, Shikoku | Okayama | Kurashiki Central Hospital | Department of General Surgery |
|  | Okayama | Okayama Saiseikai General Hospital | Departments of Surgery |
|  | Okayama | Kawasaki Medical School | Digestive Surgery |
|  | Okayama | Okayama University Graduate School of Medicine | Department of Gastroenterological Surgery |
|  | Okayama | Chugoku Rosai Hospital | Surgery |
|  | Hiroshima | National Hospital Organization Fukuyama Medical Center | Department of Surgery |
|  | Hiroshima | Hiroshima Prefectural Hospital | Gastroenterology Center |
|  | Hiroshima | National Hospital Organization Kure Medical Center and Chugoku Cancer Center | Surgery |
|  | Hiroshima | Fukuyama city hospital | Department of Digestive Surgery |
|  | Hiroshima | Hiroshima City Hiroshima Citizens Hospital | Department of Surgery |
|  | Hiroshima | JA Hiroshima General Hospital | Department of Surgery |
|  | Hiroshima | JA Onomichi General Hospital | Department of Surgery |
|  | Shimane | Shimane Prefectural Central Hospital | Department of Surgery |
|  | Yamaguchi | Yamaguchi University Graduate School of Medicine | Department of Gastroenterological, Breast and Endocrine Surgery |
|  | Yamaguchi | Iwakuni Clinical Center | Department of Surgery |
|  | Kagawa | Kagawa University | Department of Gastroenterological Surgery |
|  | Tokushima | Institute of Biochemical Sciences, Tokushima University Graduate School | Department of Digestive and Pediatric Surgery |
|  | Ehime | Uwajima City Hospital | Department of Surgery |
|  | Ehime | Matsuyama Red Cross Hospital | Department of Surgery |
|  | Ehime | Ehime University | Department of HBP and Breast Surgery |
|  | Kochi | Kochi Health Sciences Center | Department of Gastroenterological Surgery |
|  | Kochi | Kochi Medical School | Department of Surgery 1 |
| Kyushu | Fukuoka | National Hospital Organization Kyushu Cancer Center | The Department of Gastroenterological Surgery |
|  | Fukuoka | School of Medicine, University of Occupational and Environmental Health, Japan | Department of Surgery 1. |
|  | Fukuoka | Fukuoka University Hospital | Gastroenterological Surgery |
|  | Fukuoka | Graduate School of Medical Sciences, Kyushu University | Department of Surgery and Oncology |
|  | Fukuoka | Graduate School of Medical Sciences, Kyushu University | Department of Surgery and Science |
|  | Fukuoka | Fukuoka City Hospital | Department of Surgery |
|  | Fukuoka | Kurume University | Department of Surgery |
|  | Oita | Oita Red Cross Hospital | Department of Surgery |
|  | Oita | Oita University Faculty of Medicine | Department of Gastroenterological and Pediatric Surgery |
|  | Oita | National Hospital Organization Beppu Medical Center | Department of Gastroenterological Surgery |
|  | Nagasaki | Sasebo City General Hospital | Department of Gastroenterological Surgery |
|  | Nagasaki | Nagasaki University Graduate School of Biomedical Sciences | Department of Surgery |
|  | Kumamoto | Graduate School of Medical Sciences, Kumamoto University | Department of Gastroenterological Surgery |
|  | Kumamoto | Japanese Red Cross Kumamoto Hospital | Department of Surgery |
|  | Kumamoto | Saiseikai Kumamoto Hospital | Department of Surgery |
|  | Kumamoto | Kumamoto Regional Medical Center | Department of Surgery |
|  | Kumamoto | Coloproctology Center Takano Hospital | Department of Surgery |
|  | Miyazaki | University of Miyazaki Faculty of Medicine | Department of Surgery |
|  | Kagoshima | Kagoshima University School of Medicine | Department of Digestive Surgery, Breast and Thyroid Surgery |
| Total number | | 158 Institutions | 165 Departments |
